# Work Exposures and Development of Cardiovascular Diseases: A Systematic Review

**DOI:** 10.1093/annweh/wxac004

**Published:** 2022-03-03

**Authors:** Christian Moretti Anfossi, Magdalena Ahumada Muñoz, Christian Tobar Fredes, Felipe Pérez Rojas, Jamie Ross, Jenny Head, Annie Britton

**Affiliations:** Department of Epidemiology and Public Health, University College London, 1-19 Torrington Place, London, UK; Instituto de Salud Pública de Chile, Av. Marathon 1000, Santiago, Chile; Facultad de Ciencias de la salud, Campus Los Leones, Universidad San Sebastián, Lota 2465, Providencia, Santiago, Chile; Universidad Mayor sede Temuco, Av. Alemania 281, Temuco, Chile; Department of Primary Care and Population Health, University College London, London, UK; Department of Epidemiology and Public Health, University College London, 1-19 Torrington Place, London, UK; Department of Epidemiology and Public Health, University College London, 1-19 Torrington Place, London, UK

**Keywords:** cardiovascular disease, effort–reward imbalance, job insecurity, job strain, long working hours, occupational noise, shift work, systematic review

## Abstract

**Introduction:**

Cardiovascular diseases (CVDs) are the number one cause of death, and there is evidence that work exposures could be associated with their development. This study aimed to systematically review observational studies of adults exposed to job strain, effort–reward imbalance, long working hours, job insecurity, shift work, and occupational noise, and assess the association of those work exposures with CVDs.

**Methods:**

The Navigation Guide framework was applied. The population were adults of working age (18–65), and cohort and case–control studies were included. The work exposures were job strain, effort–reward imbalance, long working hours, job insecurity, shift work, and occupational noise. The outcomes were cerebrovascular diseases, ischaemic heart disease, and hypertensive diseases. The selection, data extraction, risk of bias assessment, and quality assessment were carried out by two reviewers independently and disagreements were solved by a third reviewer or by consensus. The synthesis of the results was done by applying the ‘vote counting based on direction’ method, and the results were summarized in an effect direction plot. The strength of the evidence for every risk factor and CVD was defined by consensus.

**Results:**

A total of 17 643 papers were initially identified in the literature search, but after applying the filters by title and abstract, and full text, 86 studies were finally included. From the included studies, sufficient evidence was found of the harmfulness of job strain for cerebrovascular disease and ischemic heart disease. Furthermore, there was sufficient evidence of the harmfulness of shift work for ischemic heart disease. Evidence of no relationship was found between long working hours and shift work with ischaemic heart disease and hypertensive disease, respectively. The other associations of work exposures and CVDs had limited or inadequate evidence of harmfulness.

**Conclusions:**

In this comprehensive review, there was sufficient evidence of a harmful relationship between job strain, shift work, and CVDs. For the other work exposures, more high-quality studies are needed. In order to improve current prevention strategies for CVDs, the findings of this review imply that job strain and shift work are work exposures that constitute additional risk factors that could be approached as targets for worksite interventions.

**Systematic review registration:**

PROSPERO CRD42020179972.

**What’s Important About This Paper?**

Although there are many studies on the effects of work exposures on cardiovascular diseases (CVDs), their association is still debated. This systematic review explores the association between six work exposures and three groups of CVDs described in 86 cohort and case–control studies using novel systematic review methods. This review provides identified job strain and shift work as targets for worksite interventions to prevent CVDs.

## Introduction

Cardiovascular diseases (CVDs), defined by the World Health Organization (WHO) as a ‘group of disorders of the heart and blood vessels’, are the number one cause of death globally, representing 32% of all global deaths in 2019 (WHO, 2021). This high disease prevalence has a significant economic impact on nations, but also on employers since CVDs are the most costly to companies in terms of lost productivity as a result of disability and death (International Labour Office, 2012).

Several lifestyle behaviours are associated with the development and clinical manifestation of CVDs (Neylon *et al.*, 2013). The main ones are an unhealthy diet, physical inactivity, tobacco use, and harmful use of alcohol (WHO, 2021). However, behavioural risk factors by themselves do not fully explain the population burden of CVDs (Neylon *et al.*, 2013).

The global population has experienced rapid economic growth that has resulted in a variety of occupational exposures that pose a risk for health (Gatchel and Schultz, 2012). During the 19th century, the ‘hygienist’ approach stated that the effects of work on health were due only to the lifestyle and habits of workers and the problems of urban insalubrity, taking away all the responsibility of companies (Neffa, 2015). Nowadays, the development of international policies in human, social, and labour rights has exposed the importance of working conditions and work exposures and how they represent a threat to health, safety, and well-being (Blanch, 2011).

Previous research has shown associations between working conditions and risk of CVDs (Gatchel and Schultz, 2012). One of the key underlying determinants of CVDs is stress (WHO, 2021) and one of the most studied sources of acute and chronic stress is work (Gatchel and Schultz, 2012). Traditionally, it was believed that stress and heart health were connected by associated behaviours; however, there are now well-described physiological pathways (Schnall *et al.*, 2016; American Heart Association, 2018) that involve the hypothalamic–pituitary–adrenocortical axis and the sympathetic–adrenal–medullary system (Schnall *et al.*, 2016; Bayes *et al.*, 2021).

To understand the relationship between work stress and CVDs, researchers have expanded the concept of work stress beyond the characteristics of the work task to encompass organizational factors, generating conceptual models/approaches that indicate risk conditions where those factors interact (Gatchel and Schultz, 2012; Kivimäki and Kawachi, 2015). There are six common work exposures related to CVDs described in the literature: job strain, effort–reward imbalance (ERI), long working hours, job insecurity (Kivimäki and Kawachi, 2015), shift work (Kervezee *et al.*, 2018b), and occupational noise (Teixeira *et al.*, 2019).

### Job strain

According to Karasek’s model (Karasek and Theorell, 1992), ‘job strain’ is the consequence of a combination of high job demands and low individual control over those demands (Neylon *et al.*, 2013; Kivimäki and Kawachi, 2015). The relationship between job strain and CVDs has been assessed in different populations and there is evidence that supports a positive association (Gatchel and Schultz, 2012; International Labour Office, 2012; Kivimäki and Kawachi, 2015). Evidence suggests that this model can predict myocardial infarction and cardiovascular mortality (Neylon *et al.*, 2013).

### Effort–reward imbalance

In Siegrist’s model (Siegrist, 2002), the mismatch between high effort, low reward, and the individuals’ exhaustive coping style leads to adverse health effects, as it violates core expectations about reciprocity and adequate exchange at work (Kivimäki and Kawachi, 2015). It has been shown that this model has a significant ability to predict CVDs (Neylon *et al.*, 2013).

### Long working hours

It is recognized that long working hours represent a danger to the health of workers and their families (International Labour Organization, 2019), including evidence to suggest that long working hours increases CVDs risk (Jeong *et al.*, 2013; Kivimäki and Kawachi, 2015).

### Job insecurity

There is evidence that job insecurity is associated with the incidence of CVDs (Ferrie *et al.*, 2013) and cardiovascular mortality (Vahtera *et al.*, 2004). It is hypothesized that this association is partly explained by poorer socioeconomic circumstances and less favourable risk factor profiles among people with job insecurity (Kivimäki and Kawachi, 2015).

### Shift work

Shift work, defined as ‘work occurring outside typical daytime working hours’, is associated with an increased risk of diseases (Zhao *et al.*, 2019), such as CVDs (Kervezee *et al.*, 2018b). Night shift work produces a misalignment of the endogenous circadian timing system, which is associated with alterations in a wide range of physiological parameters risky for CVDs (Kervezee et al., 2018a, b).

### Occupational noise

There is evidence that suggests that occupational noise impacts morbidity and mortality from CVDs (Babisch, 2011; Teixeira *et al.*, 2019). Exposure to certain levels of noise can lead to biochemical, physiological, and psychosocial alterations, interfering with the gastro-enteric system, endocrine system, central nervous system, and psychological alterations (Tomei *et al.*, 2010), all of them related directly or indirectly to the pathogenesis of CVDs.

Although there are many studies on the association of work exposures and CVDs, the causal connection is still subject to debate and poorly understood (Kivimäki and Kawachi, 2015; American Heart Association, 2018). Furthermore, most reviews only focus on one kind of risk factor and one disease as the outcome and include just one type of study (cohort or case–control).

It is estimated that interventions in the workplace could reduce health care costs by 26% and reduce workers’ compensation and disability management claims by 30% (Arena *et al.*, 2013); however, optimal program delivery models have yet to be elucidated. Therefore, there is a need for additional research in this area (Arena *et al.*, 2013).

To achieve a better synchronization between public policies and scientific research, solid methods to evaluate the available scientific evidence are indispensable, and currently, systematic reviews are an essential source of evidence for decision-makers (Campbell *et al.*, 2020).

This study aimed to synthesize the evidence about the association of work exposures and CVDs by performing a systematic review of observational studies of adults exposed to job strain, ERI, long working hours, job insecurity, shift work, and occupational noise to assess the association of these exposures and the development of cerebrovascular disease, ischaemic heart disease, or hypertensive disease.

## Methods

**Figure 1.**
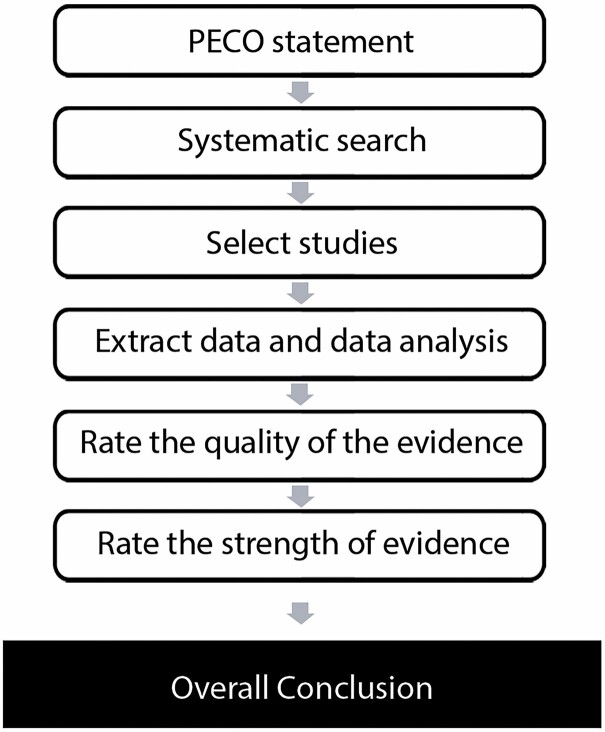
Navigation guide steps. Adapted from Woodruff and Sutton (2014).

Due to the complexity and ethical considerations, the best standard in study designs for exposures in environmental and occupational health are observational studies (Woodruff and Sutton, 2014). This constitutes an issue if systematic review methodologies such as Cochrane and the Grading of Recommendations Assessment, Development, and Evaluation (GRADE) system are to be applied because those methodologies have been developed based on randomized controlled clinical trials of interventions, considering observational studies to be ‘low-quality evidence’ (Woodruff and Sutton, 2014). As such, this systematic review has been carried out following the Navigation Guide framework, which is a systematic and rigorous approach to research synthesis, based on the best practices in the evaluation of information in evidence-based medicine and environmental health to define the strength of the evidence of toxicity or harmfulness of an exposure for specific outcomes (Woodruff and Sutton, 2014) (see Fig. 1). This framework assigns a ‘moderate’ quality rating to observational studies and allows a combination of diverse evidence streams (Woodruff and Sutton, 2014).

The protocol of this systematic review has been registered on the International Prospective Register of Systematic Reviews (PROSPERO) under the registration number CRD42020179972.

### Eligibility criteria for study selection

The study question was specified using the Population, Exposure, Comparators and Outcomes (PECO) framework (Woodruff and Sutton, 2014). A summary of the PECO statement of this study is in Table 1.

**Table 1.** PECO statement.

PECO Element	Evidence
Population	Adults in working age
Exposure	Work exposures
Comparator	Unexposed group
Outcomes	CVDs

#### Types of studies

Quantitative observational research studies, specifically cohort studies and case–control studies, were included.

Cross-sectional studies, randomized controlled trials, quasi-experimental trials, cross-over controlled trials, controlled trials without randomization, single-case studies, review articles, short communications, letters with insufficient information to analyse the results, guidelines, dissertations, qualitative studies, scientific conference abstracts, and studies on animals were excluded.

#### Population

Inclusion: Studies of adults of working age at the baseline (18–65 years), working in the formal economy.
Exclusion: Studies of children (aged <18 years), unpaid domestic workers, individuals with previous CVDs.

No restrictions were imposed on the setting of recruitment.

#### Exposures

The work exposures that have been included are job strain, ERI, long working hours, job insecurity, shift work, and occupational noise.

Studies were included if they applied descriptions of the work risk factors according to the definitions and measures in Table 2.

**Table 2.** Definitions and measures of each work exposure.

Working condition/risk factor	Definition	Measure^a^	Variable operationalization
Job strain	According to the Karasek’s model, job strain is the consequence of a combination of high job demand and low individual control over those demands (Neylon *et al.*, 2013; Kivimäki and Kawachi, 2015)	Job Content Questionnaire (Madsen *et al.*, 2017)/Demand–Control Questionnaire (Kivimäki *et al.*, 2015) and their adaptations in different languages	Combination of high demands and low control (four-quadrant diagram) (Karasek *et al.*, 1998)
ERI	In the Siegrist’s model, ERI is the mismatch between high effort, low reward, and the individuals’ exhaustive coping style (or overcommitment) (Kivimäki and Kawachi, 2015)	Effort–Reward Imbalance Questionnaire (Siegrist *et al.*, 2004) and tits adaptations in different languages	Effort–reward ratio beyond 1.0 (Siegrist *et al.*, 2004)
Long working hours	An average working time for each 7 days period over 48 h, including overtime (European Commission, n.d.)	‘Measures of the total number of hours worked, including in both of: main and secondary jobs, self-employment and salaried employment and informal and formal jobs’ (Li *et al.*, 2018)	≥48 working hours per week (European Commission, n.d.). Studies that considered more weekly hours were also included.
Job insecurity	‘The discrepancy between the level of job security a person experiences and the level she or he might prefer’ (Bartley and Ferrie, 2001)	Subscale of the Job Content Questionnaire (Job Insecurity Scale section) (Karasek *et al.*, 1998). Other questionnaires.	Perceived job insecurity (Knesebeck, 2016)
Shift work	Shift work is defined as ‘work occurring outside typical daytime working hours’ (Kervezee *et al.*, 2018b)	Work schedule (Zhao *et al.*, 2019)	Regular evening shift, regular night or graveyard shift, rotating shifts, split shifts, irregular schedule, on-call schedule, regular weekend work (Zhao *et al.*, 2019)
Occupational noise	‘Occupational noise is the exposure at the workplace to an unpleasant or unwanted sound’ (Teixeira *et al.*, 2019)	Noise measurements performed into work environments with dosimeter (Tomei *et al.*, 2010)	≥85 dB(A) (Tomei *et al.*, 2010)

^a^Studies that measured these factors using a different measure were excluded.

#### Comparator

Unexposed group.

#### Outcomes

Development of CVD according to the definitions in Supplementary File 1 (available at *Annals of Occupational Hygiene* online), which follows the International Classification of Diseases, 11th Revision, 2019 (ICD11) and its equivalence in the 10th Revision (ICD10), within the following groups of diseases: cerebrovascular diseases, ischaemic heart disease, and hypertensive diseases.

CVDs with strong congenital evidence and diseases secondary to others were excluded. Diseases of intracranial, extracranial, and coronary arteries have been excluded because the focus of this review was placed on their effects, which are included in our list of cerebrovascular diseases and ischaemic heart diseases.

For eligibility, only studies that measured CVDs with medical records were included. Self-report measures were not considered to avoid bias. Specifically for hypertension, the criteria included only studies where the diagnosis was done after two or more measures on different days to avoid confusion between hypertensive disease and ambulatory hypertension.

### Search strategy

Following the Navigation Guide recommendations, the first author conducted comprehensive literature searches using electronic academic databases for potentially relevant records from published and unpublished studies.

For published studies, the following electronic academic databases were used: Ovid Embase (1947 to 26 May 2020), Ovid Medline (1946 to 26 May 2020), PubMed (1946 to 26 May 2020), Scopus (1788 to 26 May 2020), Web of Science (1900 to 26 May 2020), and Ovid APA PsycInfo (1806 to 26 May 2020) (see Supplementary File 2 [available at *Annals of Occupational Hygiene* online]: Search Strategy in Ovid Embase as an example).

For unpublished studies, these electronic gray literature databases were used: OpenGrey (http://www.opengrey.eu/), Open Thesis (http://www.openthesis.org), Grey Literature Report (http://greylit.org/), and ProQuest Dissertations & Theses Global™ (www.proquest.co.uk/products_pq/descriptions/pqdt.shtml).

Furthermore, studies by hand searches from the following sources were included: reference lists of included papers, citing reference searching of included papers, and collections of the review authors.

The searches were conducted in English words but without a language filter. If an article was written in a language other than English or Spanish, the document was translated into one of these languages.

The downloaded references were stored in the reference managers Endnote (Beard and Aghassibakes, 2021) and Mendeley (Elston, 2019).

### Study selection

The web-based software platform Covidence (Babineau, 2014) was used to support all the stages of the systematic review. Duplicates papers were identified and deleted. One reviewer screened the titles and abstracts of the studies retrieved during the searches for relevance. Then, two reviewers assessed independently the full texts of articles identified as being potentially eligible for inclusion against the predefined criteria. Any discrepancies were resolved by consensus or by a third reviewer.

If the full text of the article was not available in the databases used, a more extensive search on the Internet was conducted. If after that search the full text was not found, the authors were contacted. References where full text and contact information were not available after the extensive search were excluded.

### Data extraction

Data were extracted by two reviewers independently and any discrepancies were resolved by consensus or by a third reviewer. The extraction was completed using a data extraction form (see Supplementary File 3 [available at *Annals of Occupational Hygiene* online]: Data Extraction Form), which was designed and piloted by the reviewers on 10 references before the data extraction.

### Data analysis

**Figure 2.**
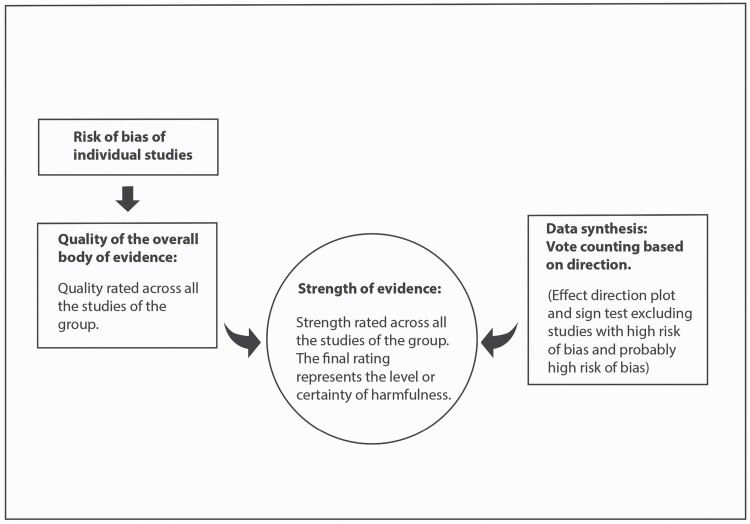
Methodology for the data analysis.

To draw conclusions about the association (harmfulness) of the work exposures and the CVDs assessed, the steps represented in Fig. 2 were applied.

#### Risk of bias of individual studies

The risk of bias of each study was assessed by two reviewers working in parallel by applying an adaptation of the Navigation Guide tool (Lam et al., in preparation; Li *et al.*, 2018) (see Supplementary File 4 [available at *Annals of Occupational Hygiene* online]: Instructions for Making Risk of Bias Determinations), which judges the risk of bias by nine domains: recruitment strategy, blinding, exposure assessment, outcome assessment, confounding (at least adjusted for age, sex and socioeconomic status), incomplete outcome data, selective outcome reporting, conflict of interest, and other sources of bias.

Every domain was graded as ‘Low Risk’, ‘Probably Low Risk’, ‘Probably High Risk’, ‘High Risk’, or ‘Not Applicable’. The worst rating in any bias domain for any outcome defined the overall risk of bias at study level (Teixeira *et al.*, 2019). Disagreements were solved by consensus or by a third reviewer.

#### Quality of the overall body of evidence

For grading the quality of evidence of each outcome by risk factor, the Navigation Guide tool (Supplementary File 5 [available at *Annals of Occupational Hygiene* online]: Instructions for Grading the Quality and Strength of Evidence) was used, which assigns a ‘moderate’ quality rating to observational studies and downgrades or upgrades the quality considering eight categories: quality of study limitations (risk of bias of individual studies), indirectness of evidence, inconsistency of evidence, imprecision of evidence, publication bias, large magnitude of effect, dose–response, and confounding minimizes the effect.

The quality of the evidence was assessed by two reviewers working in parallel using the Navigation Guide quality of evidence assessment tool, grading the evidence in ‘high’, ‘moderate’, and ‘low’. Disagreements were solved by consensus or by a third reviewer.

#### Data synthesis: vote counting based on direction

Due to the diversity in the populations and exposures in the studies, the synthesis of the data was done by a synthesis without meta-analysis (SWiM) (Campbell *et al.*, 2020).

The studies were grouped by exposure and sub-divided by outcomes. To synthesize direction of the effects for each outcome, the ‘vote counting based on direction’ method (McKenzie and Brennan, 2019) was applied. Following the recommendations of this method, a standardized binary metric was created, where each effect estimate was categorized as showing the harm or benefit based on the observed direction of effect alone (not the statistical significance of the original results of each study). If a study reported no effect or conflicting findings, this was considered as evidence in support of no association between the exposure and outcome, following the Cochrane recommendations (Higgins *et al.*, 2019).

The number of studies (votes) showing harm were compared with the number showing benefit using a sign test excluding those with no effect or conflicting findings (McKenzie and Brennan, 2019). The results of the ‘vote counting based on direction’ method and the sign tests have been summarized in an effect direction plot (Fig. 4) (Boon and Thomson, 2021). For the sign test, the website GraphPad (https://www.graphpad.com/quickcalcs/binomial1/) was used to calculate the one-tailed *P*-value for each outcome domain (Boon and Thomson, 2021). Studies with ‘high risk of bias’ and ‘probably high risk of bias’ were excluded from the effect direction plot, but they were considered to draw conclusions about the strength of the evidence (Fig. 2).

#### Strength of evidence

Finally, the overall strength of the body of evidence of each outcome by working condition and risk factor was rated based on four considerations following the Navigation Guide criteria: quality of body of evidence, direction of effect, confidence in effect, and other compelling attributes of the data that may influence certainty.

The final decision about the strength of the evidence was done by the reviewers by consensus by applying the definitions in Supplementary File 6 (available at *Annals of Occupational Hygiene* online): Instructions for Grading the Quality and Strength of Evidence. The strength of the evidence was rated in one of the following four categories: sufficient evidence of harmfulness, limited evidence of harmfulness, inadequate evidence of harmfulness, and evidence of lack of harmfulness.

Although the statistical significance of the results of the individual studies was not considered for the effect direction plot, this information was an input for the final decisions.

## Results

**Figure 3.**
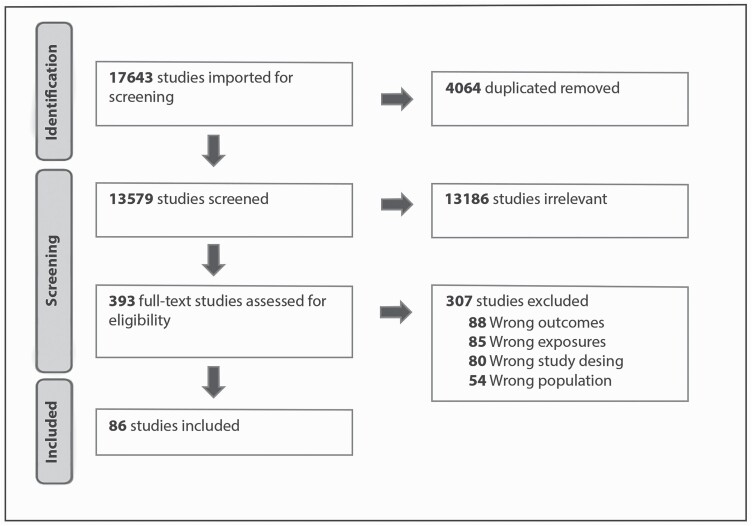
PRISMA flow diagram.

A total of 17 643 papers were identified in the literature search, 4064 duplicated records were detected and removed by Covidence and manually, leaving 13 579. After reading the titles and abstracts, 393 studies were selected for full-text screening. Of those, 86 studies were included (see Fig. 3).

The included 86 studies were published between 1982 and 2020. Studies from North America, Europe, and Asia were included; no studies from Africa, South America, or Australia passed the filters. Of the 86 studies, 22 were case–control and 64 were cohort studies.

For the analysis, studies were separated and grouped by exposures and CVD outcome. Studies with more than one exposure or/and more than one CVD (outcome) were counted again in their respective groups, raising the number of studies in every group by exposure and CVD from 86 to 114.

Distribution of studies by risk factor:

• ERI: 6 studies
• Job insecurity: 8 studies
• Job strain: 40 studies
• Long working hours: 17 studies
• Occupational noise: 7 studies
• Shift work: 36 studies

Distribution of studies by outcome:

• Cerebrovascular disease: 21 studies
• Hypertensive disease: 22 studies
• Ischaemic heart disease: 71 studies

**Figure 4.**
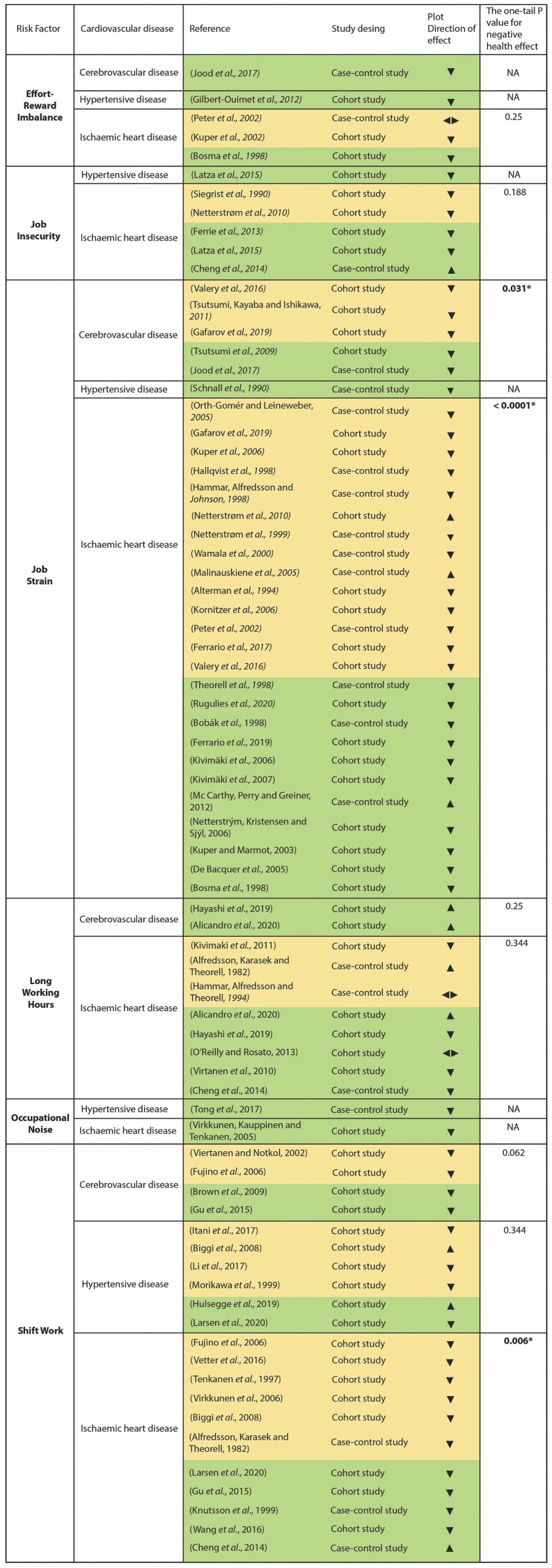
Effect direction plot. Upward arrow (▲) = positive heath effect (negative relationship between work exposure and CVD); downward arrow (▼) = negative health effect (positive relationship between work exposure and CVD); sideways arrow ◄ ► = no change/mixed effects/conflicting findings. Set arrow size (large, medium, small) to reflect sample size: ▲ > 300; ▲ 50–300; ▲ < 50. Yellow = probably low risk of bias; green = low risk of bias. *P-value statistically significant (*P*-value <0.05). NA = sign test not applicable because just one study was included.

In the Effect Direction Plot, papers with a high and probably high risk of bias were excluded, incorporating 75 of the 114 studies (Fig. 4).

### Effort–reward imbalance

For the association of ERI and cerebrovascular diseases, one case–control study with a low risk of bias was included (Fig. 4). The overall quality of the evidence was upgraded to ‘high quality’ because all the important confounders were considered. The final decision about the strength of the evidence was ‘limited evidence of harmfulness’ (Table 3) because a positive relationship was observed between exposure and outcome with statistical significance and low risk of bias, however, confidence in the relationship is constrained by the number of studies (just one).

**Table 3.** Strength of evidence by exposure and CVD.

		Strength of the evidence			
Exposure	Outcome	Sufficient evidence of harmfulness	Limited evidence of harmfulness	Inadequate evidence of harmfulness	Evidence of lack of harmfulness
ERI	Cerebrovascular disease		X		
	Hypertensive disease		X		
	Ischaemic heart disease		X		
Job insecurity	Cerebrovascular disease			X	
	Hypertensive disease		X		
	Ischaemic heart disease		X		
Job strain	Cerebrovascular disease	X			
	Hypertensive disease			X	
	Ischaemic heart disease	X			
Long working hours	Cerebrovascular disease			X	
	Hypertensive disease			X	
	Ischaemic heart disease				X
Occupational noise	Cerebrovascular disease			X	
	Hypertensive disease		X		
	Ischaemic heart disease		X		
Shift work	Cerebrovascular disease		X		
	Hypertensive disease				X
	Ischaemic heart disease	X			

X, final decision about the strength of the evidence.

For hypertensive diseases, two cohort studies were included, one with a low risk of bias (Fig. 4) and one with a probably high risk of bias (Supplementary File 6 [available at *Annals of Occupational Hygiene* online]: Data Summary). The overall quality of evidence was moderate. The strength of the evidence of harmfulness was limited (Table 3), because a positive relationship was observed between exposure and outcomes in both studies, but with statistical significance just for women ≥45 years old in one of them. The confidence in the relationship is also constrained by the number of studies.

One case–control and two cohort studies were included for ERI and ischaemic heart disease, two had a probably low risk of bias and one had a low risk of bias (Fig. 4). The overall quality of evidence was ‘high quality’ because all the important confounders were considered in 67% of the studies. The strength of the evidence was ‘limited evidence of harmfulness’ (Table 3) due to the reduced number of studies (for a significant sign test result) (Fig. 4).

### Job insecurity

Only one cohort study with a probably high risk of bias passed the filters for job insecurity and cerebrovascular disease (Supplementary File 6 [available at *Annals of Occupational Hygiene* online]: Data Summary) and therefore the strength of the evidence was ‘inadequate evidence of harmfulness’ (Table 3). This study showed no effect of job insecurity as a risk factor for cerebrovascular diseases, but it was deemed insufficient to assess the effect of the exposure, due to the reduced number of studies and the risk of bias.

For hypertensive disease, one cohort study with a low risk of bias was included (Fig. 4). The strength of the evidence was ‘limited evidence of harmfulness’ (Table 3) because even when a positive relationship was observed between exposure and outcome with statistical significance and moderate overall quality, confidence in the relationship is constrained by the number of studies (just one).

Five cohort studies and one case–control study were included for job insecurity and ischaemic heart disease, five with a low or probably low risk of bias (Fig. 4) and one with a probably high risk of bias (Supplementary File 6 [available at *Annals of Occupational Hygiene* online]: Data Summary). The overall quality of the evidence was high because all the important confounders were considered in most of the studies, and they showed similar results for the same exposure. The strength of the evidence was ‘limited evidence of harmfulness’ (Table 3) because a positive association was observed in most of the included studies (most of them without statistical significance) (Supplementary File 6 [available at *Annals of Occupational Hygiene* online]: Data Summary), but the confidence in the relationship is constrained by chance due to the reduced number of studies (for a significant sign test result) (Fig. 4).

### Job strain

In the group of studies of job strain and cerebrovascular disease, one case–control study and seven cohort studies were included. Five studies had a low or probably low risk of bias (Fig. 4) and three had a probably high risk of bias (Supplementary File 6 [available at *Annals of Occupational Hygiene* online]: Data Summary). The overall quality of the evidence was upgraded as ‘high quality’, due to the consistency in the results across the studies. The strength of the evidence was defined as ‘sufficient evidence of harmfulness’ (Table 3) because a positive relationship was observed between exposure and outcome where chance can be ruled out with reasonable confidence (sign test *P*-value 0.0313) (Fig. 4) with high-quality evidence.

Two case–control studies and one cohort study were included to assess the association of job strain and hypertensive disease. One study had a low risk of bias (Fig. 4) and two had a high or probably high risk of bias (Supplementary File 6 [available at *Annals of Occupational Hygiene* online]: Data Summary). The overall quality of the evidence was downgraded to ‘low quality’ due to the individual risk of bias of two of the studies included. The strength of the evidence was rated as ‘inadequate evidence of harmfulness’ (Table 3) due to the limited number of studies and the low quality of the overall evidence.

In the group of studies of the association of job strain and ischaemic heart disease, 10 case–control studies and 19 cohort studies were included. Twenty-five studies had a low or probably low risk of bias (Fig. 4), three had a probably high risk of bias and one had a high risk of bias (Supplementary File 6 [available at *Annals of Occupational Hygiene* online]: Data Summary). The overall quality of the evidence was upgraded to ‘high quality’ because 59% of studies considered all the important confounders and 90% of the studies considered at least one important confounder. The final decision for the strength of the evidence was ‘sufficient evidence of harmfulness’ (Table 3) because a positive relationship was observed between job strain and ischaemic heart disease and chance could be ruled out with reasonable confidence (sign test *P*-value < 0.0001) (Fig. 4), with high-quality evidence.

### Long working hours

For long working hours and cerebrovascular disease, one case–control study and three cohort studies were included. Two studies had a low risk of bias (Fig. 4) and two studies had a probably high risk of bias (Supplementary File 6 [available at *Annals of Occupational Hygiene* online]: Data Summary). The overall quality of the evidence was downgraded to ‘low quality’ due to the individual risk of bias of the studies and the inconsistency in the results across the studies. The final decision of the strength of the evidence was ‘inadequate evidence of harmfulness’ (Table 3) because the available evidence was insufficient to assess effects of the exposure due to the limited number of studies, the low quality of the evidence, and the mixed results.

The included papers for long working hours and hypertensive disease were two cohort studies, both with a high risk of bias (Supplementary File 6 [available at *Annals of Occupational Hygiene* online]: Data Summary), the reason why the overall quality of the evidence was downgraded to ‘low quality’. The strength of the evidence was ‘inadequate evidence of harmfulness’ (Table 3) because of the limited number of studies, the low quality of individual studies, and the inconsistency of findings across individual studies.

Five case–control studies and six cohort studies were included for long working hours and ischaemic heart disease. Eight studies had a low or probably low risk of bias (Fig. 4), and three had a high or probably high risk of bias (Supplementary File 6 [available at *Annals of Occupational Hygiene* online]: Data Summary). The quality of the evidence was moderate, and the final decision of the strength of the evidence was ‘evidence of lack of harmfulness’ (Table 3), because no consistency of a negative effect was observed across the studies with low and probably low risk of bias after applying the sign test (Fig. 4). Furthermore, from the total 11 studies included, just one has statistical significance for exposure over 48 h per week (our initial definition of long working hours). Three studies had statistical significance for exposure over 55 h per week and one for exposure over 50.

### Occupational noise

Just one cohort study with a probably high risk of bias was included for occupational noise and cerebrovascular disease (Supplementary File 6 [available at *Annals of Occupational Hygiene* online]: Data Summary), and therefore the quality of the evidence was downgraded to ‘low’. The strength of the evidence was decided as ‘inadequate evidence of harmfulness’, because of the limited number of studies and the low quality of the evidence.

For hypertensive disease, one case–control study and two cohort studies were included. One had a probably low risk of bias (Fig. 4), and two a probably high risk of bias (Supplementary File 6 [available at *Annals of Occupational Hygiene* online]: Data Summary). The overall quality was rated as ‘low’. The strength of the evidence was valued as ‘limited evidence of harmfulness’ (Table 3) because a positive relationship was observed with statistical significance but just three studies were included and two with probably high risk of bias.

Three cohort studies were included to assess the association of occupational noise and ischaemic heart disease. One had a probably low risk of bias (Fig. 4) and two a probably high risk of bias (Supplementary File 6 [available at *Annals of Occupational Hygiene* online]: Data Summary), the reason why the overall quality of the evidence was downgraded to ‘low quality’. The strength of the evidence was defined as ‘limited evidence of harmfulness’ (Table 3). A positive relationship was observed, but chance, bias, and confounding cannot be ruled out with reasonable confidence.

### Shift work

For the association of shift work and cerebrovascular disease, six cohort studies were included, four of them had a low or probably low risk of bias (Fig. 4) and two had a probably high risk of bias (Supplementary File 6 [available at *Annals of Occupational Hygiene* online]: Data Summary). The overall quality of the evidence was upgraded to high quality because the studies reported similar results for the same exposure and most of them considered the important confounders. The strength of the evidence was rated as ‘limited evidence of harmfulness’ (Table 3) because a positive relationship was observed between exposure and outcome, but chance cannot be ruled out with a sign test due to the number of studies (Fig. 4).

Eleven cohort studies and one case–control study were included for shift work and hypertensive disease, six had a low or probably low risk of bias (Fig. 4) and six had a high or probably high risk of bias (Supplementary File 6 [available at *Annals of Occupational Hygiene* online]: Data Summary). The overall quality of the evidence was moderate. The strength was rated as ‘evidence of lack of harmfulness’ (Table 3), because no consistency of a negative effect was observed across the studies with low and probably low risk of bias after applying the sign test (Fig. 4). Additionally, from the 12 studies included, 42% of the studies have no statistical significance of a positive relationship and one study has statistical significance for a positive health effect.

For shift work and ischaemic heart disease, 4 case–control studies and 14 cohort studies were included. Eleven studies had a low or probably low risk of bias (Fig. 4) and seven had a high or probably high risk of bias (Supplementary File 6 [available at *Annals of Occupational Hygiene* online]: Data Summary). The overall quality was upgraded to ‘high’ because most of the studies (56%) included all the important confounders. The final decision on the strength of the evidence was ‘sufficient evidence of harmfulness’ (Table 3) because a positive relationship was observed between shift work and ischaemic heart disease where chance, bias, and confounding can be ruled out with reasonable confidence (high-quality evidence and *P*-vale sign test 0.0059) (Fig. 4).

## Discussion

In this review, 86 relevant observational studies were identified. Sufficient evidence of harmfulness was found between job strain and cerebrovascular disease, job strain and ischaemic heart disease, and shift work and ischemic heart disease. In contrast, no evidence of a harmful relationship was observed between long working hours and shift work with ischaemic heart disease and hypertensive disease, respectively. These groups of exposures and CVDs include results from well-designed, well-conducted studies. The other associations of this review were classified with limited or inadequate evidence of harmfulness (Table 3), which means that more high-quality studies are needed to draw conclusions.

For the general structure of this systematic review, we applied the Navigation Guide framework. This methodology is a novel alternative for systematic reviews in occupational and environmental health where randomized controlled trials of potentially harmful exposures are not possible. The Navigation Guide assigns *a priori* a ‘moderate’ quality rating to the body of human observational evidence, which differs from other methodologies for systematic reviews in the clinical sciences, such as GRADE, which assign them *a priori* a ‘low’ quality (Woodruff and Sutton, 2014). This review only included observational studies; therefore, the tools in the Navigation Guide were important in rating the quality of individual studies and the quality of the overall body of evidence.

Due to the clinical and methodological diversity, and the different effect measures of the studies, for data synthesis, we applied the vote counting method and an effect direction plot, following the latest recommendations of Cochrane (Higgins *et al.*, 2019; Boon and Thomson, 2021). However, when a sign test is also applied, it is necessary to have at least five studies to rule out chance in the results across the studies. This was not possible for some of our groups of exposures and CVDs. In those groups of associations with more than one study, no methodological or effect measure diversity, and no substantial heterogeneity, a meta-analysis could provide valuable information.

In accordance with our results, other reviews applying meta-analysis found significant associations between job strain and CVDs (Niedhammer *et al.*, 2021), and shift work and CVDs (Vyas *et al.*, 2012; Wang *et al.*, 2018). Another exposure widely studied is long working hours (Niedhammer *et al.*, 2021); however, most of the reviews considered long working over our limit of ≥48 h/week (Table 2) (e.g. over 55 h/week) (Virtanen and Kivimäki, 2018). In a systematic review and meta-analysis of only cohort studies (Kivimäki *et al.*, 2015), it was estimated that 49–54 working hours per week had a relative risk (RR) of 1.27 (1.03–1.56) compared with standard working hours.

While governments are primarily responsible for public health policies, employers have a direct responsibility to provide a safe and hazard-free environment for workers (Tarro *et al.*, 2020), because, as is known, exposures in the workplace could enhance or harm physical and mental health (Zusman *et al.*, 2021). Considering that budgets for health are limited, decisions about public policy and occupational medicine should be made based on the best evidence available, which is just achievable through systematics reviews with robust methods. Currently, systematic reviews that apply the Cochrane methods and tools, including a meta-analysis are the best standard; however, these are not always feasible in systematic reviews of observational studies of work exposures that include different study designs and measures of effect, so this study provides a combination of alternative methods for those cases.

Regarding CVDs, current strategies mainly focused on controlling the cardiovascular factors by individuals’ health care providers have not been enough to reduce the rising prevalence of these diseases (Padwal *et al.*, 2017). Therefore, this review provides pivotal evidence in identifying the potential for job strain and shift work as additional risk factors to focus on since according to our results, from the multiple exposures assessed, they showed sufficient evidence of harm to the development of CVDs. These findings are relevant for approaches that suggest that worksite interventions could be a suitable alternative to reduce cardiovascular risks (Arena *et al.*, 2013) with job strain and shift work as possible targets for those interventions.

Our results concur with previous reviews, but our findings were corroborated by applying an approach specifically created for systematic reviews of environmental and work exposures, and by presenting results of varied studies in a way other than a narrative synthesis. Though there are several studies about work exposures and CVDs, our findings from multiple exposures also confirmed that more high-quality evidence is needed.

## Supplementary Material

wxac004_suppl_Supplementary_File_1

wxac004_suppl_Supplementary_File_2

wxac004_suppl_Supplementary_File_3

wxac004_suppl_Supplementary_File_4

wxac004_suppl_Supplementary_File_5

wxac004_suppl_Supplementary_File_6

## Data Availability

The data underlying this article will be shared on reasonable request to the corresponding author.
