# Supplementary material for: Work Exposures and Development of Cardiovascular Diseases: A Systematic Review
Source: Ann Work Expo Health. 2022 Mar 3;66(6):698–713. doi: 10.1093/annweh/wxac004 (PMC9250287; doi:10.1093/annweh/wxac004)
Supplement: wxac004_suppl_Supplementary_File_1 [file wxac004_suppl_supplementary_file_1.docx]

**Supplementary file 1**

**Work exposures and development of cardiovascular diseases: A systematic review.**

**CHRISTIAN MORETTI ANFOSSI^1^*, MAGDALENA AHUMADA MUÑOZ^2^, CHRISTIAN TOBAR FREDES^3^, FELIPE PÉREZ ROJAS^4^, JAMIE ROSS^5^ JENNY HEAD^1^, ANNIE BRITTON^1^.**

*^1^University College London, Department of Epidemiology and Public Health, 1-19 Torrington Place, London WC1E 7HB, United Kingdom; ^2^Instituto de Salud Pública de Chile, Av. Marathon 1000, Santiago de Chile; ^3^Universidad San Sebastián,* *Facultad de Ciencias de la salud, Campus Los Leones, Santiago, Chile; ^4^Universidad Mayor sede Temuco, Av. Alemania 281, Temuco, Chile. ^5^University College London, Department of Primary Care and Population Health, Rowland Hill Street, London NW3 2PF, United Kingdom*

***** Author to whom correspondence should be addressed. Tel: +44 7 999070843; e-mail: christian.anfossi.19@ucl.ac.uk

**Inclusion criteria for diseases (International Classification of Diseases 11/10th Revisions, 2019)** (*ICD-11 - Mortality and Morbidity Statistics*, no date)**.**

| Group of diseases (Outcomes) | Diseases included | ICD11 Codes | ICD10 Codes |
| --- | --- | --- | --- |
| Cerebrovascular diseases | Intracranial haemorrhage | 8B00-8B03, 8B0Z | I60-I69 |
|  | Cerebral ischaemia | 8B10, 8B11, 8B1Y, 8B1Z | G45, I63, I67.8, I67.8 |
|  | Stroke not known if ischaemic or haemorrhagic | 8B20 | I64 |
|  | Cerebrovascular disease with no acute cerebral symptom | 8B21 | I60-I69 |
|  | Certain specified cerebrovascular diseases | 8B22 | I67 |
|  | Hypoxic-ischaemic encephalopathy | 8B24 | G93.1 |
|  | Cerebrovascular diseases, unspecified | 8B2Z | I60-I69 |
| Ischaemic heart disease | Acute ischaemic heart disease | BA40-BA43, BA4Z | I20-I25 |
|  | Chronic ischaemic heart disease | BA50, BA51, BA5Z | I25.2, I25.5, I25 |
|  | Ischaemic heart diseases, unspecified | BA6Z | I20-I25 |
| Hypertensive diseases | Essential hypertension | BA00 | I10 |
|  | Hypertensive heart disease | BA01 | I11 |

For studies that applied older revisions of the International Classification of Diseases, the equivalences of the diseases were used according to the WHO references.
