# Supplementary material for: Work Exposures and Development of Cardiovascular Diseases: A Systematic Review
Source: Ann Work Expo Health. 2022 Mar 3;66(6):698–713. doi: 10.1093/annweh/wxac004 (PMC9250287; doi:10.1093/annweh/wxac004)
Supplement: wxac004_suppl_Supplementary_File_2 [file wxac004_suppl_supplementary_file_2.docx]

**Supplementary file 2:**

**Work exposures and development of cardiovascular diseases: A systematic review.**

**CHRISTIAN MORETTI ANFOSSI^1^*, MAGDALENA AHUMADA MUÑOZ^2^, CHRISTIAN TOBAR FREDES^3^, FELIPE PÉREZ ROJAS^4^, JAMIE ROSS^5^ JENNY HEAD^1^, ANNIE BRITTON^1^.**

*^1^University College London, Department of Epidemiology and Public Health, 1-19 Torrington Place, London WC1E 7HB, United Kingdom; ^2^Instituto de Salud Pública de Chile, Av. Marathon 1000, Santiago de Chile; ^3^Universidad San Sebastián,* *Facultad de Ciencias de la salud, Campus Los Leones, Santiago, Chile; ^4^Universidad Mayor sede Temuco, Av. Alemania 281, Temuco, Chile. ^5^University College London, Department of Primary Care and Population Health, Rowland Hill Street, London NW3 2PF, United Kingdom*

***** Author to whom correspondence should be addressed. Tel: +44 7 999070843; e-mail: christian.anfossi.19@ucl.ac.uk

**Search Strategy in Ovid Embase:**

Exposure:

1. job satisfaction/ or psychometry/ or Job Content Questionnaire.mp. or job stress/
2. mental stress/ or job strain.mp.
3. psychology/ or job demand*.mp. or workload/
4. job control.mp.
5. reward/ or Effort reward imbalance.mp.
6. Effort-reward imbalance.mp.
7. Effort-reward balance.mp.
8. effort reward balance.mp.
9. Overtime Work*.mp.
10. work schedule/ or Overwork*.mp. or workload/
11. working time/ or Long working hour*.mp.
12. job overload*.mp.
13. work overload*.mp.
14. job security/ or Job Insecurity.mp.
15. unemployment/
16. shift worker/ or shift* work*.mp. or night shift/
17. shift* job*.mp.
18. night work/ or Night* work*.mp.
19. motivation/ or Evening* work*.mp. or evening shift/
20. Night* shift*.mp.
21. Night-shift*.mp.
22. Evening* shift*.mp.
23. Weekend* work*.mp.
24. Weekend* shift*.mp.
25. irregular Schedule*.mp.
26. Regular evening shift.mp.
27. regular night.mp.
28. personnel management/ or graveyard shift*.mp.
29. rotating shift*.mp.
30. split shift*.mp.
31. irregular schedule*.mp.
32. on-call schedule*.mp.
33. working time/ or regular weekend work*.mp.
34. Noise, Occupational.mp. or industrial noise/
35. Occupational Noise*.mp. or occupational exposure/
36. Work Noise*.mp.
37. Noise labour*.mp.
38. Noise labor*.mp.

Outcome:

1. Cardiovascular Diseases.mp. or cardiovascular disease/
2. Cardiovascular System.mp. or cardiovascular system/
3. Cardiovascular.mp. or cardiovascular function/ or cardiovascular mortality/ or cardiovascular effect/ or cardiovascular risk/
4. Cerebrovascular Disorders.mp.
5. intracranial embolism.mp. or brain embolism/
6. intracranial thrombosis.mp. or occlusive cerebrovascular disease/
7. cardioembolic stroke/ or stroke*.mp.
8. Brain Diseases, Metabolic/ or Brain Disease*, Metabolic.mp.
9. ischemic heart disease/ or Ischaemic heart disease*.mp.
10. intracranial hemorrhages.mp. or brain hemorrhage/
11. cerebral hemorrhage.mp.
12. subarachnoid hemorrhage.mp. or subarachnoid hemorrhage/
13. Nontraumatic subdural haemorrhage.mp.
14. Brain Ischemia.mp. or brain ischemia/
15. Cerebral Infarction.mp. or brain infarction/
16. ischemic attack, transient.mp. or transient ischemic attack/
17. Heart attack.mp. or heart infarction/
18. stroke/ or Cerebral ischaemic stroke.mp. or cerebrovascular accident/
19. Cerebral ischaemia*.mp.
20. Cerebral ischemia*.mp.
21. Hypoxic-ischaemic encephalopathy.mp. or hypoxic ischemic encephalopathy/
22. Hypoxia-Ischemia, Brain.mp.
23. Myocardial Ischemia.mp. or heart muscle ischemia/
24. Ischemic cardiomyopathy.mp. or ischemic heart disease/ or ischemic cardiomyopathy/
25. Coronary thrombosis.mp. or coronary artery thrombosis/
26. Coronary Heart Disease*.mp.
27. Angina Pectoris.mp. or angina pectoris/
28. Myocardial Infarction.mp.
29. Anterior Wall Myocardial Infarction.mp. or anterior myocardial infarction/
30. Inferior Wall Myocardial Infarction.mp. or inferior myocardial infarction/
31. heart infarction/ or Subsequent myocardial infarction.mp. or acute heart infarction/
32. Subsequent myocardial infarction.mp.
33. Chronic ischaemic heart disease, unspecified.mp.
34. Myocardial Ischemia.mp. or heart muscle ischemia/
35. Essential Hypertension.mp. or essential hypertension/
36. hypertension.mp. or hypertension/
37. Hypertensive crisis.mp. or hypertensive crisis/ or blood pressure/

Type of Study:

1. observational study.mp. or observational study/
2. cohort study.mp. or cohort analysis/
3. case control study/ or case control.mp.
4. retrospective study/ or retrospective.mp.
5. longitudinal.mp. or longitudinal study/
6. nonexperimental.mp.
7. non-experimental.mp.
8. nonrandomized.mp.
9. nonrandomised.mp.
10. non-randomized.mp.
11. non-randomised.mp.

Combinations:

1. 1 or 2 or 3 or 4 or 5 or 6 or 7 or 8 or 9 or 10 or 11 or 12 or 13 or 14 or 15 or 16 or 17 or 18 or 19 or 20 or 21 or 22 or 23 or 24 or 25 or 26 or 27 or 28 or 29 or 30 or 31 or 32 or 33 or 34 or 35 or 36 or 37 or 38
2. 49 or 50 or 51 or 52 or 53 or 54 or 55 or 56 or 57 or 58 or 59 or 60 or 61 or 62 or 63 or 64 or 65 or 66 or 67 or 68 or 69 or 70 or 71 or 72 or 73 or 74 or 75
3. 76 or 77 or 78 or 79 or 80 or 81 or 82 or 83 or 84 or 85 or 86
4. 87 and 88 and 89
