# Supplementary material for: Work Exposures and Development of Cardiovascular Diseases: A Systematic Review
Source: Ann Work Expo Health. 2022 Mar 3;66(6):698–713. doi: 10.1093/annweh/wxac004 (PMC9250287; doi:10.1093/annweh/wxac004)
Supplement: wxac004_suppl_Supplementary_File_3 [file wxac004_suppl_supplementary_file_3.docx]

# **Supplementary File 3:**

**Work exposures and development of cardiovascular diseases: A systematic review.**

**CHRISTIAN MORETTI ANFOSSI^1^*, MAGDALENA AHUMADA MUÑOZ^2^, CHRISTIAN TOBAR FREDES^3^, FELIPE PÉREZ ROJAS^4^, JAMIE ROSS^5^ JENNY HEAD^1^, ANNIE BRITTON^1^.**

*^1^University College London, Department of Epidemiology and Public Health, 1-19 Torrington Place, London WC1E 7HB, United Kingdom; ^2^Instituto de Salud Pública de Chile, Av. Marathon 1000, Santiago de Chile; ^3^Universidad San Sebastián,* *Facultad de Ciencias de la salud, Campus Los Leones, Santiago, Chile; ^4^Universidad Mayor sede Temuco, Av. Alemania 281, Temuco, Chile. ^5^University College London, Department of Primary Care and Population Health, Rowland Hill Street, London NW3 2PF, United Kingdom*

***** Author to whom correspondence should be addressed. Tel: +44 7 999070843; e-mail: christian.anfossi.19@ucl.ac.uk

# **Data Extraction Form.**

| Study ID |  |
| --- | --- |
| Title |  |
| Reviewer Name |  |
| Year (publication) |  |
| Lead author name |  |
| Email |  |
| Address |  |
| Country in which the study conducted |  |
| Notes |  |
| Aim of study |  |
| Study design |  |
| Start date |  |
| End date |  |
| Length of participant follow up |  |
| Single or Multicenter |  |
| Study funding sources |  |
| Possible conflicts of interest for study authors |  |
| Likelihood of reporting other biases |  |
| Methods used to prevent and address missing data |  |
| Unit of analysis |  |
| Age |  |
| Sex |  |
| Inclusion criteria |  |
| Exclusion criteria |  |
| Method of recruitment of participants |  |
| Total number of participants |  |
| The International Standard Classification of Occupations (ISCO) |  |
| Industrial Setting |  |
| Risk Factor 1 |  |
| - Measure of exposure |  |
| - Outcome (Risk Factor 1) |  |
| - Description of the comparison/control group |  |
| - Incidence |  |
| - Cumulative Incidence Ratio CI (95%) |  |
| - Prevalence |  |
| - Prevalence ratio |  |
| - Confounders in the model |  |
| - Relative Risk CI (95%) |  |
| - Odds Ratio CI (95%) |  |
| - Hazard Ratio CI (95%) |  |
| - Coefficient of correlation |  |
| - Regression |  |
| - Attributable risk in exposed |  |
| - Population attributable risk |  |
| - Attributable fraction in exposed |  |
| - Population attributable fraction |  |
| - Other (Name) |  |
| - Result of "Other" |  |
| - Direction of effect (+, 0, -) |  |
| Risk Factor 2 |  |
| - Measure of exposure |  |
| - Outcome (Risk Factor 2) |  |
| - Description of the comparison/control group |  |
| - Incidence |  |
| - Cumulative Incidence Ratio CI (95%) |  |
| - Prevalence |  |
| - Prevalence ratio |  |
| - Confounders in the model |  |
| - Relative Risk CI (95%) |  |
| - Odds Ratio CI (95%) |  |
| - Hazard Ratio CI (95%) |  |
| - Coefficient of correlation |  |
| - Regression |  |
| - Attributable risk in exposed |  |
| - Population attributable risk |  |
| - Attributable fraction in exposed |  |
| - Population attributable fraction |  |
| - Other (Name) |  |
| - Result of "Other" |  |
| - Direction of effect (+, 0, -) |  |
| Risk Factor 3 |  |
| - Measure of exposure |  |
| - Outcome (Risk Factor 3) |  |
| - Description of the comparison/control group |  |
| - Incidence |  |
| - Cumulative Incidence Ratio CI (95%) |  |
| - Prevalence |  |
| - Prevalence ratio |  |
| - Confounders in the model |  |
| - Relative Risk CI (95%) |  |
| - Odds Ratio CI (95%) |  |
| - Hazard Ratio CI (95%) |  |
| - Coefficient of correlation |  |
| - Regression |  |
| - Attributable risk in exposed |  |
| - Population attributable risk |  |
| - Attributable fraction in exposed |  |
| - Population attributable fraction |  |
| - Other (Name) |  |
| - Result of "Other" |  |
| - Direction of effect (+, 0, -) |  |
| Risk Factor 4 |  |
| - Measure of exposure |  |
| - Outcome (Risk Factor 4) |  |
| - Description of the comparison/control group |  |
| - Incidence |  |
| - Cumulative Incidence Ratio CI (95%) |  |
| - Prevalence |  |
| - Prevalence ratio |  |
| - Confounders in the model |  |
| - Relative Risk CI (95%) |  |
| - Odds Ratio CI (95%) |  |
| - Hazard Ratio CI (95%) |  |
| - Coefficient of Correlation |  |
| - Regression |  |
| - Attributable risk in exposed |  |
| - Population attributable risk |  |
| - Attributable fraction in exposed |  |
| - Population attributable fraction |  |
| - Other (Name) |  |
| - Result of "Other" |  |
| - Direction of effect (+, 0, -) |  |
| Key conclusions of the study authors |  |
