# Supplementary material for: Work Exposures and Development of Cardiovascular Diseases: A Systematic Review
Source: Ann Work Expo Health. 2022 Mar 3;66(6):698–713. doi: 10.1093/annweh/wxac004 (PMC9250287; doi:10.1093/annweh/wxac004)
Supplement: wxac004_suppl_Supplementary_File_4 [file wxac004_suppl_supplementary_file_4.docx]

**Supplementary file 4:**

**Work exposures and development of cardiovascular diseases: A systematic review.**

**CHRISTIAN MORETTI ANFOSSI^1^*, MAGDALENA AHUMADA MUÑOZ^2^, CHRISTIAN TOBAR FREDES^3^, FELIPE PÉREZ ROJAS^4^, JAMIE ROSS^5^ JENNY HEAD^1^, ANNIE BRITTON^1^.**

*^1^University College London, Department of Epidemiology and Public Health, 1-19 Torrington Place, London WC1E 7HB, United Kingdom; ^2^Instituto de Salud Pública de Chile, Av. Marathon 1000, Santiago de Chile; ^3^Universidad San Sebastián,* *Facultad de Ciencias de la salud, Campus Los Leones, Santiago, Chile; ^4^Universidad Mayor sede Temuco, Av. Alemania 281, Temuco, Chile. ^5^University College London, Department of Primary Care and Population Health, Rowland Hill Street, London NW3 2PF, United Kingdom*

***** Author to whom correspondence should be addressed. Tel: +44 7 999070843; e-mail: christian.anfossi.19@ucl.ac.uk

**Instructions for Making Risk of Bias Determinations**

The instructions and criteria for judging the risk of bias in this tool have been adapted from the Case Study #7 of application of the Navigation Guide Systematic Review Methodology (1), therefore, most of the text has been adopted verbatim from that study. In some specific points we include some of the adaptations done by the WHO and the ILO to the same tool in their latest protocols to assess occupational risks (2).

**Human Studies**

**Please answer LOW RISK, PROBABLY LOW RISK, PROBABLY HIGH RISK, HIGH RISK or NOT APPLICABLE and provide details/justification.**

Note: These criteria for judging risk of bias are for human studies only since we are not evaluating animal studies in this study.

**1. Was the strategy for recruiting participants consistent across study groups?**

**Criteria for a judgment of LOW risk of bias (i.e., answer: “YES”):**

Protocols for recruitment and inclusion/exclusion criteria were applied similarly across study groups, and any one of the following:

- Study participants were recruited from the same population at the same time frame; or
- Study participants were not all recruited from the same population, but proportions of participants from each population in each study group are uniform

**Criteria for the judgment of PROBABLY LOW risk of bias (i.e., answer: “Probably Yes”):**

There is insufficient information about participant selection to permit a judgment of ‘YES’, but there is indirect evidence that suggests that participant recruitment and inclusion/exclusion criteria was consistent, as described by the criteria for a judgment of ‘YES’.

**Criteria for the judgment of HIGH risk of bias (i.e., answer: “No”):**

Any of the following:

- Protocols for recruitment or inclusion/exclusion criteria were applied differently across study groups; or
- Study participants were recruited at different time frames; or
- Study participants were recruited from different populations and proportions of participants from each population in each study group are not uniform
- Differential loss to follow‐up between groups
- Reported refusal/non‐response is uniform between groups

**Criteria for the judgment of PROBABLY HIGH risk of bias (i.e., answer: “Probably No”):**

There is insufficient information about participant selection to permit a judgment of ‘NO’, but there is indirect evidence that suggests that participant recruitment or inclusion/exclusion criteria was inconsistent, as described by the criteria for a judgment of ‘NO’.

**Criteria for the judgment of NOT APPLICABLE (risk of bias domain is not applicable to study):**

There is evidence that participant selection is not an element of study design capable of introducing risk of bias in the study.

**2. Was knowledge of the exposure adequately prevented during the study?**

**Criteria for a judgment of LOW risk of bias (i.e., answer: “YES”):**

Any of the following:

- No blinding, but the review authors judge that the outcome and the outcome measurement as well as the exposure and exposure measurement are not likely to be influenced by lack of blinding (such as differential outcome assessment where the outcome is assessed using different measurement or estimation metrics across exposure groups, or differential exposure assessment where exposure is assessed using different measurement or estimation metrics across diagnostic or outcome groups); or
- Blinding of key study personnel was ensured, and it is unlikely that the blinding could have been broken; or
- Some key study personnel were not blinded, but exposure and outcome assessment was blinded and the non‐blinding of others is unlikely to introduce bias. For example, investigators were effectively blinded to the exposure and/or outcome groups, for example if the exposure was measured by a separate entity and the outcome was obtained from a hospital record.

**Criteria for the judgment of PROBABLY LOW risk of bias (i.e., answer: “Probably Yes”):**

There is insufficient information about blinding to permit a judgment of low risk of bias, but there is indirect evidence that suggests the study was adequately blinded, as described by the criteria for a judgment of low risk of bias.

**Criteria for the judgment of HIGH risk of bias (i.e., answer: “No”):**

Any of the following:

- No blinding or incomplete blinding, and the outcome or outcome measurement or exposure and exposure measurement is likely to be influenced by lack of blinding (i.e., differential outcome or exposure assessment); or
- Blinding of key study personnel attempted, but likely that the blinding could have been broken so as to introduce bias; or
- Some key study personnel were not blinded, and the non‐blinding of others was likely to introduce bias.

**Criteria for the judgment of PROBABLY HIGH risk of bias (i.e., answer: “Probably No”):**

There is insufficient information about blinding to permit a judgment of high risk of bias, but there is indirect evidence that suggests the study was not adequately blinded, as described by the criteria for a judgment of high risk of bias.

**Criteria for the judgment of NOT APPLICABLE (risk of bias domain is not applicable to study):**

There is evidence that blinding is not an element of study design capable of introducing risk of bias in the study.

**3. Were exposure assessment methods robust?**

Note: For this risk of bias domain, we will consider exposure assessment according to our definitions in **Additional File 2**.

The following list of considerations represents a collection of factors proposed may potentially influence the internal validity of the exposure assessment in a systematic manner (not those that may randomly affect overall study results). These should be interpreted only as suggested considerations and should not be viewed as scoring or a checklist.

**List of Considerations:**

Possible sources of exposure assessment metrics:

1. Official Records (Ministry of Health, Ministry of Labour or other official sources)
2. Organization
3. Self reported
4. Combination of the above options

For each, overall considerations include:

1. What is the quality of the source of the metric being used?
2. Is the exposure measured in the study a surrogate for the exposure?
3. What was the temporal coverage (i.e. short or long-term exposure)?
4. Did the analysis account for prediction uncertainty?
5. How was missing data accounted for, and any data imputations incorporated?
6. Were sensitivity analyses performed?

In particular, for exposure assessment models:

1. Were the input data in the study suspected to systematically under- or over-estimate exposure?
2. What type of model was used?
3. What was geographic/spatial accuracy (county, census tract, organization, individual residence)?
4. What was the temporal specificity and variation?
5. What was the space-time coverage of the model?
6. Were time-activity patterns accounted for?

**Criteria for a judgment of LOW risk of bias (i.e., answer: “Yes”):**

The reviewers judge that there is low risk of exposure misclassification, i.e.:

- There is high confidence in the accuracy of the exposure assessment methods, such as methods that have been tested for validity and reliability in measuring the targeted exposure; or
- Less‐established or less direct exposure measurements are validated against well‐ established or direct methods.

**Criteria for the judgment of PROBABLY LOW risk of bias (i.e., answer: “Probably No”):**

There is insufficient information about the exposure assessment methods to permit a judgment of low risk of bias, but there is indirect evidence that suggests that methods were robust, as described by the criteria for a judgment of low risk of bias.

**Criteria for the judgment of HIGH risk of bias (i.e., answer: “No”):**

The reviewers judge that there is high risk of exposure misclassification and any one of the following:

- There is low confidence in the accuracy of the exposure assessment methods; or
- Less‐established or less direct exposure measurements are not validated and are suspected to introduce bias that impacts the outcome assessment (example: participants are asked to report exposure status retrospectively, subject to recall bias); or
- Uncertain how exposure information was obtained.

**Criteria for the judgment of PROBABLY HIGH risk of bias (i.e., answer: “Probably No”):**

There is insufficient information about the exposure assessment methods to permit a judgment of high risk of bias, but there is indirect evidence that suggests that methods were not robust, as described by the criteria for a judgment of high risk of bias.

**Criteria for the judgment of NOT APPLICABLE (risk of bias domain is not applicable to study):**

There is evidence that exposure measurement methods are not capable of introducing risk of bias in the study.

**4. Were outcome assessment methods accurate?**

**Criteria for a judgment of LOW risk of bias (i.e. answer: “Yes”):**

The reviewers judge that there is low risk of outcome misclassification, i.e.:

- Outcomes were assessed and defined consistently across all study participants, using valid and reliable measures (solid medical records). Note that all outcome assessment measures captured in the PECO statement are considered beforehand to be valid and reliable, unless other information provided within the study warrants a consideration otherwise; or
- Less-established or less direct outcome measurements are validated against well- established or direct methods; or
- Appropriate sensitivity analyses were conducted that suggest the influence of outcome misclassification would be minimal
- AND, if applicable, appropriate QA/QC for methods is described and is satisfactory.

**Criteria for the judgment of PROBABLY LOW risk of bias (i.e. answer: “Probably Yes”):**

There is insufficient information about the outcome assessment methods to permit a judgment of low risk of bias, but there is indirect evidence which suggests that methods were robust, as described by the criteria for a judgment of low risk of bias. Appropriate QA/QC for methods are not described but the review authors judge that the outcome and the outcome assessment are objective and uniform across study groups.

**Criteria for the judgment of HIGH risk of bias (i.e. answer: “No”):**

The reviewers judge that there is high risk of outcome misclassification and any one of the following:

- There is low confidence in the accuracy of the outcome assessment methods; or
- Less-established or less direct outcome measurements are not validated and are suspected to introduce bias that impacts the outcome assessment
- Uncertain how outcome information was obtained

**Criteria for the judgment of PROBABLY HIGH risk of bias (i.e. answer: “Probably No”):**

There is insufficient information about the outcome assessment methods to permit a judgment of high risk of bias, but there is indirect evidence which suggests that methods were not robust, as described by the criteria for a judgment of high risk of bias.

**Criteria for the judgment of NOT APPLICABLE (risk of bias domain is not applicable to study):**

There is evidence that outcome assessment methods are not capable of introducing risk of bias in the study.

**5. Was confounding adequately addressed?**

Prior to the evaluation of studies, co-authors collectively developed the following important confounders:

- Age, sex and socioeconomic position.

**Criteria for a judgment of LOW risk of bias (i.e., answer: “Yes”):**

The study accounted for (i.e., matched, stratified, multivariate analysis or otherwise statistically controlled for) all 3 important potential confounders, or reported that potential confounders were evaluated and omitted because inclusion did not substantially affect the results. The determination of specific confounders may be informed by the data, including the studies included in the review.

**Criteria for the judgment of PROBABLY LOW risk of bias (i.e., answer: “Probably Yes”):**

The study accounted for most but not all of the important potential confounders AND this lack of accounting is not expected to introduce substantial bias.

**Criteria for the judgment of HIGH risk of bias (i.e., answer: “No”):**

The study did not account for or evaluate multiple important confounders.

**Criteria for the judgment of PROBABLY HIGH risk of bias (i.e., answer: “Probably No”):**

The study accounted for at least one of the important potential confounders but included other potential confounders AND this lack of accounting may have introduced substantial bias.

**6. Were incomplete outcome data adequately addressed?**

**Criteria for a judgment of LOW risk of bias (i.e., answer: “Yes”):**

Participants were followed long enough to obtain outcome measurements;

OR any one of the following:

- No missing outcome data; or
- Reasons for missing outcome data unlikely to be related to true outcome (for survival data, censoring unlikely to introduce bias); or
- Attrition or missing outcome data balanced in numbers across exposure groups, with similar reasons for missing data across groups; or
- For dichotomous outcome data, the proportion of missing outcomes compared with observed event risk not enough to have a relevant impact on the intervention effect estimate; or
- For continuous outcome data, plausible effect size (difference in means or standardized difference in means) among missing outcomes not enough to have a relevant impact on the observed effect size; or
- Missing data have been imputed using appropriate methods

**Criteria for the judgment of PROBABLY LOW risk of bias (i.e. answer: “Probably Yes”):**

There is insufficient information about incomplete outcome data to permit a judgment of low risk of bias, but there is indirect evidence that suggests incomplete outcome data was adequately addressed, as described by the criteria for a judgment of low risk of bias.

**Criteria for the judgment of HIGH risk of bias (i.e., answer: “No”):**

Participants were not followed long enough to obtain outcome measurements; OR any one of the following:

- Reason for missing outcome data likely to be related to true outcome, with either imbalance in numbers or reasons for missing data across exposure groups; or
- For dichotomous outcome data, the proportion of missing outcomes compared with observed event risk enough to induce biologically relevant bias in intervention effect estimate; or
- For continuous outcome data, plausible effect size (difference in means or standardized difference in means) among missing outcomes enough to induce biologically relevant bias in observed effect size; or
- Potentially inappropriate application of imputation.

**Criteria for the judgment of PROBABLY HIGH risk of bias (i.e., answer: “Probably No”):**

There is insufficient information about incomplete outcome data to permit a judgment of high risk of bias, but there is indirect evidence that suggests incomplete outcome data was not adequately addressed, as described by the criteria for a judgment of high risk of bias.

**Criteria for the judgment of NOT APPLICABLE (risk of bias domain is not applicable to study):**

There is evidence that incomplete outcome data is not capable of introducing risk of bias in the study.

**7. Does the study report appear to have been comprehensive in its outcome reporting?**

**Criteria for a judgment of LOW risk of bias (i.e., answer: “Yes”):**

All of the study’s pre‐specified (primary and secondary) outcomes outlined in the protocol, methods, abstract, and/or introduction that are of interest in the review have been reported in the pre‐specified way.

**Criteria for the judgment of PROBABLY LOW risk of bias (i.e. answer: “Probably Yes”):**

There is insufficient information about selective outcome reporting to permit a judgment of low risk of bias, but there is indirect evidence that suggests the study was free of selective reporting, as described by the criteria for a judgment of low risk of bias.

**Criteria for the judgment of HIGH risk of bias (i.e., answer: “No”):**

Any one of the following:

- Not all of the study’s pre‐specified primary outcomes (as outlined in the protocol, methods, abstract, and/or introduction) have been reported; or
- One or more primary outcomes is reported using measurements, analysis methods or subsets of the data (e.g. subscales) that were not pre‐specified; or
- One or more reported primary outcomes were not pre‐specified (unless clear justification for their reporting is provided, such as an unexpected effect); or
- One or more outcomes of interest are reported incompletely

**Criteria for the judgment of PROBABLY HIGH risk of bias (i.e., answer: “Probably No”):**

There is insufficient information about selective outcome reporting to permit a judgment of high risk of bias, but there is indirect evidence that suggests the study was not free of selective reporting, as described by the criteria for a judgment of high risk of bias.

**Criteria for the judgment of NOT APPLICABLE (risk of bias domain is not applicable to study):**

There is evidence that selective outcome reporting is not capable of introducing risk of bias in the study.

**8. Is the study free of support from any company, study author, or other entity having a financial interest in any of the exposures studied?**

**Criteria for a judgment of LOW risk of bias (i.e., answer: “Yes”):**

The study did not receive support from a company, study author, or other entity having a financial interest in the outcome of the study. Examples include the following:

- Funding source is limited to government, non‐profit organizations, or academic grants funded by government, foundations and/or non‐profit organizations;
- Chemicals or other treatment used in study were purchased from a supplier;
- Company affiliated staff are not mentioned in the acknowledgements section;
- Authors were not employees of a company with a financial interest in the outcome of the study;
- Company with a financial interest in the outcome of the study was not involved in the design, conduct, analysis, or reporting of the study and authors had complete access to the data;
- Study authors make a claim denying conflicts of interest;
- Study authors are unaffiliated with companies with financial interest, and there is no reason to believe a conflict of interest exists;
- All study authors are affiliated with a government agency (are prohibited from involvement in projects for which there is a conflict of interest or an appearance of conflict of interest).

**Criteria for the judgment of PROBABLY LOW risk of bias (i.e. answer: “Probably Yes”):**

There is insufficient information to permit a judgment of low risk of bias, but there is indirect evidence that suggests the study was free of support from a company, study author, or other entity having a financial interest in the outcome of the study, as described by the criteria for a judgment of low risk of bias.

**Criteria for the judgment of HIGH risk of bias (i.e., answer: “No”):**

The study received support from a company, study author, or other entity having a financial interest in the outcome of the study. Examples of support include:

• Research funds;

• Chemicals, equipment or testing provided at no cost;

• Writing services;

• Author/staff from study was employee or otherwise affiliated with company with financial interest;

• Company limited author access to the data;

• Company was involved in the design, conduct, analysis, or reporting of the study;

• Study authors claim a conflict of interest

**Criteria for the judgment of PROBABLY HIGH risk of bias (i.e., answer: “Probably No”):**

There is insufficient information to permit a judgment of high risk of bias, but there is indirect evidence that suggests the study was not free of support from a company, study author, or other entity having a financial interest in the outcome of the study, as described by the criteria for a judgment of high risk of bias.

**Criteria for the judgment of NOT APPLICABLE (risk of bias domain is not applicable to study):**

There is evidence that conflicts of interest are not capable of introducing risk of bias in the study.

**9. Did the study appear to be free of other problems that could put it at a risk of bias?**

**Criteria for a judgment of LOW risk of bias (i.e., answer: “Yes”):**

The study appears to be free of other sources of bias.

**Criteria for the judgment of PROBABLY LOW risk of bias (i.e. answer: “Probably Yes”):**

There is insufficient information to permit a judgment of low risk of bias, but there is indirect evidence that suggests the study was free of other threats to validity.

**Criteria for the judgment of HIGH risk of bias (i.e., answer: “No”):**

There is at least one important risk of bias. For example, the study:

- Had a potential source of bias related to the specific study design used; or
- Stopped early due to some data‐dependent process (including a formal‐stopping rule); or
- The conduct of the study is affected by interim results (e.g. recruiting additional participants from a subgroup showing greater or lesser effect); or
- Has been claimed to have been fraudulent; or
- Had some other problem

**Criteria for the judgment of PROBABLY HIGH risk of bias (i.e., answer: “Probably No”):**

There is insufficient information to permit a judgment of high risk of bias, but there is indirect evidence that suggests the study was not free of other threats to validity, as described by the criteria for a judgment of high risk of bias.

**References:**

1. Chiu W, Johnson N, Moriarty M, Pulczinsk J, Uwak I, Taiwo S, et al. Applying the Navigation Guide Systematic Review Methodology Case Study #7: Association between Prenatal Exposures to Ambient Air Pollution and Birthweight. 2017;1–55. Available from: http://www.crd.york.ac.uk/PROSPEROFILES/17890_PROTOCOL_20150226.pdf

2. Li J, Brisson C, Clays E, Ferrario MM, Ivanov ID, Landsbergis P, et al. WHO/ILO work-related burden of disease and injury: Protocol for systematic reviews of exposure to long working hours and of the effect of exposure to long working hours on ischaemic heart disease. Environ Int [Internet]. 2018;119(August):558–69. Available from: https://doi.org/10.1016/j.envint.2018.06.022
