# Supplementary material for: Work Exposures and Development of Cardiovascular Diseases: A Systematic Review
Source: Ann Work Expo Health. 2022 Mar 3;66(6):698–713. doi: 10.1093/annweh/wxac004 (PMC9250287; doi:10.1093/annweh/wxac004)
Supplement: wxac004_suppl_Supplementary_File_5 [file wxac004_suppl_supplementary_file_5.docx]

# Supplementary file 5:

**Work exposures and development of cardiovascular diseases: A systematic review.**

**CHRISTIAN MORETTI ANFOSSI^1^*, MAGDALENA AHUMADA MUÑOZ^2^, CHRISTIAN TOBAR FREDES^3^, FELIPE PÉREZ ROJAS^4^, JAMIE ROSS^5^ JENNY HEAD^1^, ANNIE BRITTON^1^.**

*^1^University College London, Department of Epidemiology and Public Health, 1-19 Torrington Place, London WC1E 7HB, United Kingdom; ^2^Instituto de Salud Pública de Chile, Av. Marathon 1000, Santiago de Chile; ^3^Universidad San Sebastián,* *Facultad de Ciencias de la salud, Campus Los Leones, Santiago, Chile; ^4^Universidad Mayor sede Temuco, Av. Alemania 281, Temuco, Chile. ^5^University College London, Department of Primary Care and Population Health, Rowland Hill Street, London NW3 2PF, United Kingdom*

***** Author to whom correspondence should be addressed. Tel: +44 7 999070843; e-mail: [christian.anfossi.19@ucl.ac.uk](mailto:christian.anfossi.19@ucl.ac.uk)

**Instructions for Grading the Quality and Strength of Evidence**

The instructions and criteria for grading the quality and strength of evidence in this tool have been adapted from the Case Study #7 of application of the Navigation Guide Systematic Review Methodology (1), therefore, most of the text has been adopted verbatim from that study. In some specific points we include some of the adaptations done by the WHO and the ILO to the same tool in their latest protocols to assess occupational risks (2).

1. ***Grading Quality***

Each of the categories to consider in downgrading or upgrading the evidence is described in detail below. Please record your results on the chart at the end of each category, including a brief explanation for your ratings.

**Category 1. Quality of Study Limitations (Risk of Bias)**

Possible ratings: 0=no change; -1 or -2 downgrade 1 or 2 levels

The evidence from studies can be rated down if most of the relevant evidence comes from studies that suffer from a high risk of bias. Risk of bias is rated by outcome across studies. Study limitations for each outcome for individual studies and across studies are summarized in the heat maps. GRADE outlines the following principles for moving from risk of bias in individual studies to rating quality of evidence across studies.

1. In deciding on the overall quality of evidence, one does not average across studies (for instance if some studies have no serious limitations, some serious limitations, and some very serious limitations, one does not automatically rate quality down by one level because of an average rating of serious limitations). Rather, judicious consideration of the contribution of each study, with a general guide to focus on the high-quality studies is warranted.^^[[1]](#footnote-1)^^
2. This judicious consideration requires evaluating the extent to which each study contributes toward the estimate of magnitude of effect. The contribution that each study makes will usually reflect study sample size and number of outcome events. Larger studies with many events will contribute more, much larger studies with many more events will contribute much more.
3. One should be conservative in the judgment of rating down. That is, one should be confident that there is substantial risk of bias across most of the body of available evidence before one rates down for risk of bias.
4. The risk of bias should be considered in the context of other limitations. If, for instance, reviewers find themselves in a close-call situation with respect to two quality issues (risk of bias and, say, precision), GRADE suggests rating down for at least one of the two.
5. Notwithstanding the first four principles, reviewers will face close-call situations. You should acknowledge that you are in such a situation, make it explicit why you think this is the case, and make the reasons for your ultimate judgment apparent.

| **Rating for Risk of Bias (Study Limitations)**  0 no change  -1 decrease quality 1 level  -2 decrease quality 2 levels | | **Rationale for your judgment** |
| --- | --- | --- |
| Human |  |  |

**Category 2. Indirectness of Evidence**

Possible ratings: 0=no change; -1 or -2 downgrade 1 or 2 levels

Quality of evidence (your confidence in estimates of effect) may decrease when substantial differences exist between the population, exposure, or outcomes measured in the research studies under consideration in the review.

Evidence is direct when it directly compares the exposures in which we are interested in the populations in which we are interested and measures outcomes important to the study question (in GRADE the outcomes must be important to patients).

Based on GRADE, evidence can be indirect in one of three ways.^^[[2]](#footnote-2)^^

- 1. The population studied differs from the population of interest (the term applicability is often used for this form of indirectness). GRADE states that in general, one should not rate down for population differences unless one has compelling reason to think that the biology in the population of interest is so different than the population tested that the magnitude of effect will differ substantially. According to GRADE, most often, this will not be the case.
  2. The intervention (exposure) tested may differ from the exposure of interest, i.e., a difference in the chemical, route and/or dose. Decisions regarding indirectness of populations and exposure depend on an understanding of whether biological or social factors are sufficiently different that one might expect substantial differences in the magnitude of effect. GRADE also states, “As with all other aspects of rating quality of evidence, there is a continuum of similarity of the intervention that will require judgment. It is rare, and usually unnecessary, for the intended populations and interventions to be identical to those in the studies, and we should only rate down if the differences are considered sufficient to make a difference in outcome likely.”
  3. Outcomes may differ from those of primary interest; for instance, surrogate outcomes that are not themselves important, but measured in the presumption that changes in the surrogate reflect changes in an important outcome. The difference between desired and measured outcomes may relate to time frame. When there is a discrepancy between the time frame of measurement and that of interest, whether to rate down by one or two levels will depend on the magnitude of the discrepancy. Another source of indirectness related to measurement of outcomes is the use of substitute or surrogate endpoints in place of the exposed population’s important outcome of interest. In general, the use of a surrogate outcome requires rating down the quality of evidence by one, or even two, levels. Consideration of the biology, mechanism, and natural history of the disease can be helpful in making a decision about indirectness. Surrogates that are closer in the putative causal pathway to the adverse outcomes warrant rating down by only one level for indirectness. GRADE states that rarely, surrogates are sufficiently well established that one should choose not to rate down quality of evidence for indirectness. In general, evidence based on surrogate outcomes should usually trigger rating down, whereas the other types of indirectness will require a more considered judgment.

| **Rating for Indirectness**  0 no change  -1 decrease quality 1 level  -2 decrease quality 2 levels | | **Rationale for your judgment** |
| --- | --- | --- |
| Human |  |  |

**Category 3. Inconsistency of Evidence**

Possible ratings: 0 = no change; -1 or -2 downgrade 1 or 2 levels

According to Cochrane, “when studies yield widely differing estimates of effect (heterogeneity or variability in results) investigators should look for robust explanations for that heterogeneity.

…When heterogeneity exists and affects the interpretation of results, but authors fail to identify a plausible explanation, the quality of the evidence decreases.”

Based on GRADE, **a body of evidence is not rated up in quality if studies yield consistent results, but may be rated down in quality if inconsistent.** Their stated reason is that a consistent bias will lead to consistent, spurious findings.

GRADE suggests rating down the quality of evidence if large inconsistency (heterogeneity) in study results remains after exploration of a priori hypotheses that might explain heterogeneity. Judgment of the extent of heterogeneity is based on similarity of point estimates, extent of overlap of confidence intervals, and statistical criteria. GRADE’s recommendations refer to inconsistencies in effect size, specifically to relative measures (risk ratios and hazard ratios or odds ratios), not absolute measures.

Based on GRADE, reviewers should consider rating down for inconsistency when:

1. Point estimates vary widely across studies;
2. Confidence intervals (CIs) show minimal or no overlap;
3. The statistical test for heterogeneity-which tests the null hypothesis that all studies in a meta- analysis have the same underlying magnitude of effect- shows a low P-value;
4. The I*^2^* -which quantifies the proportion of the variation in point estimates due to among-study differences-is large. (I.e., the I^2^ index quantifies the degree of heterogeneity in a meta-analysis).

GRADE states that inconsistency is important **only when it reduces confidence in results in relation to a particular decision.** Even when inconsistency is large, it may not reduce confidence in results regarding a particular decision. For example, studies that are inconsistent related to the magnitude of a beneficial or harmful effect (but are in the same direction) would not be rated down; in instances when results are inconsistent as to whether there is a benefit or harm of treatment, GRADE would rate down the quality of evidence as a result of variability in results, because the meaning of the inconsistency is so relevant to the decision to treat or not to treat.

| **Rating for Inconsistency**  0 no change  -1 decrease quality 1 level  -2 decrease quality 2 levels | | **Rationale for your judgment** |
| --- | --- | --- |
| Human |  |  |

**Category 4. Imprecision of Evidence**

Possible ratings: 0=no change; -1 or -2 downgrade 1 or 2 levels

Cochrane states that when studies have few participants and few events, and thus have wide confidence intervals (CIs), authors can lower their rating of the quality of evidence. These ratings of precision are made as judgments by review authors. The ratings are made by looking across studies, or, if available, on the results of a meta-analysis.

GRADE defines evidence quality differently for systematic reviews and guidelines. For systematic reviews, quality refers to confidence in the estimates of effect. For guidelines, quality refers to the extent to which confidence in the effect estimate is adequate to support a particular decision. For the purpose of step 3 of *Navigation Guide*, we will use the systematic review definition, because the decision phase does not occur until step 4 when recommendations for prevention are made. Thus, when reviewing the data for imprecision, evaluate your confidence in the estimate of the effect.

According to GRADE, to a large extent, CIs inform the impact of random error on evidence quality. Thus, when considering imprecision, the issue is whether the CI around the estimate of exposure effect is sufficiently narrow. If it is not, GRADE rates down the evidence quality by one level (for instance, from high to moderate). If the CI is very wide, GRADE might rate down by two levels.

| **Rating for Imprecision**  0 no change  -1 decrease quality 1 level  -2 decrease quality 2 levels | | **Rationale for your judgment** |
| --- | --- | --- |
| Human |  |  |

**Category 5. Publication Bias**

Possible ratings: 0 = no change; -1 or -2 downgrade 1 or 2 levels

GRADE and Cochrane assess publication bias in a similar manner. Whereas “selective outcome reporting” is assessed for each study included in the review as part of the risk of bias assessment, “publication bias” is assessed on the body of evidence. GRADE states that “when an entire study remains unreported and the results relate to the size of the effect- publication bias- one can assess the likelihood of publication bias only by looking at a group of studies.”

Cochrane’s definition of publication bias is “the *publication* or *non-publication* of research findings depending on the nature and direction of the results.” Cochrane and GRADE are primarily concerned with *overestimates* of true effects of treatments or pharmaceuticals, especially related to “small studies effects”, i.e., the tendency for estimates of an intervention to be more beneficial in smaller studies. There is empirical evidence in the clinical sciences that publication and other reporting biases result in over estimating the effects of interventions.

In contrast, in environmental health, we are primarily concerned with *underestimating* the true effects of a chemical exposure, since in many cases population wide exposure has already occurred. We are also concerned that studies finding no association are less likely to be published because journals are less likely to publish “negative” findings.

Applying this inverted concern to GRADE’s assessment for publication bias, leads to these considerations when rating publication bias:

- - Early *negative* studies, particularly if small in size, are suspect. (GRADE is concerned with early *positive* studies).
  - Authors of systematic reviews should suspect publication bias when studies are uniformly small, particularly when sponsored by the industry. (Same as GRADE)
  - Empirical examination of patterns of results (e.g., funnel plots) may suggest publication bias but should be interpreted with caution. (Same as GRADE)
  - More compelling than any of these theoretical exercises is authors’ success in obtaining the results of some unpublished studies and demonstrating that the published and unpublished data show different results. (Same as GRADE)
  - Comprehensive searches of the literature including unpublished studies, i.e., the grey literature, and a search for research in other languages are important to addressing publication bias. Note that Cochrane also states “comprehensive searching is not sufficient to prevent some substantial potential biases.”

| **Rating for Publication Bias**  0 no change  -1 decrease quality 1 level  -2 decrease quality 2 levels | | **Rationale for your judgment** |
| --- | --- | --- |
| Human |  |  |

**Upgrade Categories**

GRADE states that the circumstances for upgrading likely occur infrequently and are primarily relevant to observational and other non-randomized studies. Although it is possible to rate up results from randomized controlled trials, GRADE has yet to find a compelling circumstance for doing so.

GRADE specifies 3 categories for increasing the quality of evidence.

**Category 6. Large Magnitude of Effect**

Possible ratings: 0 = no change; +1 or +2 upgrade 1 or 2 levels

Modelling studies suggests that confounding (from non-random allocation) alone is unlikely to explain associations with a relative risk (RR) greater than 2 (or less than 0.5), and very unlikely to explain associations with an RR greater than 5 (or less than 0.2). Thus, these are the definitions of “large magnitude of effect” used by GRADE to upgrade 1 or 2 levels, respectively. Also, GRADE is more likely to rate up if the effect is rapid and out of keeping with prior trajectory; usually supported by indirect evidence. GRADE presents empirical evidence to support these conclusions, and states that “although further research is warranted, both modelling and empirical work suggest the size of bias from confounding is unpredictable in direction but bounded in size.

Hence, the GRADE group has previously suggested guidelines for rating quality of evidence up by one category (typically from low to moderate) for associations greater than 2, and up by two categories for associations greater than 5.”

Applying the GRADE definitions of large magnitude of effect i.e., RR greater than 2 or 5 is problematic in environmental health because for dichotomous outcomes RR is a function of the exposure comparator; these definitions also are not applicable to results from continuous variables. At present, we do not have an empirically defined “large magnitude of effect.” Therefore, for the purpose of this case study, review authors should assess whether the results indicate a large magnitude of effect using their expert judgment of “large effects” in environmental health and state their definition for discussion by the group.

| **Rating for Large Magnitude of Effect**  0 no change  +1 increase quality 1 level  +2 increase quality 2 levels | | **Rationale for your judgment** |
| --- | --- | --- |
| Human |  |  |

**Category 7. Dose-response**

Possible ratings: 0 = no change; +1 or +2 upgrade 1 or 2 levels

Possible considerations include consistent dose response gradients in one or multiple studies, and/or dose response across studies, depending on the overall relevance to the body of evidence.

| **Rating for Dose-Response**  0 no change  +1 increase quality 1 level  +2 increase quality 2 levels | | **Rationale for your judgment** |
| --- | --- | --- |
| Human |  |  |

**Category 8. Confounding Minimizes Effect**

Possible ratings: 0=no change; +1 or +2 upgrade 1 or 2 levels

All plausible residual confounders or biases would reduce a demonstrated effect, or suggest a spurious effect when results show no effect.

| **Rating for Confounding Minimizes Effect**  0 no change  +1 increase quality 1 level  +2 increase quality 2 levels | | **Rationale for your judgment** |
| --- | --- | --- |
| Human |  |  |

The results of the reviewers’ ratings by population will be compiled and discussed leading to a final decision on overall quality of human evidence. The rationale for the decision will be fully documented.

**Final decision on overall quality of human evidence:**

(Example: Moderate quality is upgraded 1 step to high for XYZ reason(s))

---- High

---- Moderate

---- Low

1. ***Rate the Strength of Evidence***

The evidence quality ratings will be translated into strength of evidence for each population based on a combination of four criteria: (1) Quality of body of evidence; (2) Direction of effect; (3) Confidence in effect; and (4) Other compelling attributes of the data that may influence certainty. The strength of evidence ratings are summarized in Table 1 below, where their meaning is further defined.

**Table 1. Strength of evidence definitions for human evidence**

Strength Rating Definition

| Sufficient  evidence of harmfulness | A positive relationship is observed between exposure and outcome where chance, bias, and confounding can be ruled out with reasonable confidence. The available evidence includes results from one or more well-designed, well-conducted studies, and the conclusion is unlikely to be strongly affected by the results of future studies. |
| --- | --- |
| Limited  Evidence of harmfulness | A positive relationship is observed between exposure and outcome where chance, bias, and confounding cannot be ruled out with reasonable confidence. Confidence in the relationship is constrained by such factors as: the number, size, or quality of individual studies, or inconsistency of findings across individual studies. As more information becomes available,  the observed effect could change, and this change may be large enough to alter the conclusion. |
| Inadequate  Evidence of harmfulness | The available evidence is insufficient to assess effects of the exposure. Evidence is insufficient because of: the limited number or size of studies, low quality of individual studies, or inconsistency of findings across individual studies. More information may allow an assessment of effects. |
| Evidence of Lack of harmfulness | No relationship is observed between exposure and outcome, and chance, bias and confounding can be ruled out with reasonable confidence. The available evidence includes consistent results from more than one well-designed, well-conducted study at the full range of exposure levels that humans are known to encounter, and the conclusion is unlikely to be strongly affected by the results of future studies. The conclusion is limited to the age at exposure and/or other  conditions and levels of exposure studied. |

**References**

1. Chiu W, Johnson N, Moriarty M, Pulczinsk J, Uwak I, Taiwo S, et al. Applying the Navigation Guide Systematic Review Methodology Case Study #7: Association between Prenatal Exposures to Ambient Air Pollution and Birthweight. Prep [Internet]. 2017;1–55. Available from: http://www.crd.york.ac.uk/PROSPEROFILES/17890_PROTOCOL_20150226.pdf

2. Li J, Brisson C, Clays E, Ferrario MM, Ivanov ID, Landsbergis P, et al. WHO/ILO work-related burden of disease and injury: Protocol for systematic reviews of exposure to long working hours and of the effect of exposure to long working hours on ischaemic heart disease. Environ Int [Internet]. 2018;119(August):558–69. Available from: https://doi.org/10.1016/j.envint.2018.06.022

1. a Note: Limitations to GRADE’s risk of bias assessments as stated by GRADE: “First, empirical evidence supporting the criteria is limited. Attempts to show systematic difference between studies that meet and do not meet specific criteria have shown inconsistent results. Second, the relative weight one should put on the criteria remains uncertain. The GRADE approach is less comprehensive than many systems, emphasizing simplicity and parsimony over completeness. GRADE’s approach does not provide a quantitative rating of risk of bias. Although such a rating has advantages, we share with the Cochrane Collaboration methodologists a reluctance to provide a risk of bias score that, by its nature, must make questionable assumptions about the relative extent of bias associated with individual items and fails to consider the context of the individual items.” [↑](#footnote-ref-1)
2. GRADE includes a fourth type of indirectness that occurs when there are no direct (i.e., head-to-head) comparisons between two or more interventions of interest. This criterion is not relevant to our study question. [↑](#footnote-ref-2)
