# Supplementary material for: Work Exposures and Development of Cardiovascular Diseases: A Systematic Review
Source: Ann Work Expo Health. 2022 Mar 3;66(6):698–713. doi: 10.1093/annweh/wxac004 (PMC9250287; doi:10.1093/annweh/wxac004)
Supplement: wxac004_suppl_Supplementary_File_6 [file wxac004_suppl_supplementary_file_6.docx]

**Supplementary File 6:**

**Work exposures and development of cardiovascular diseases: A systematic review.**

**CHRISTIAN MORETTI ANFOSSI^1^*, MAGDALENA AHUMADA MUÑOZ^2^, CHRISTIAN TOBAR FREDES^3^, FELIPE PÉREZ ROJAS^4^, JAMIE ROSS^5^ JENNY HEAD^1^, ANNIE BRITTON^1^.**

*^1^University College London, Department of Epidemiology and Public Health, 1-19 Torrington Place, London WC1E 7HB, United Kingdom; ^2^Instituto de Salud Pública de Chile, Av. Marathon 1000, Santiago de Chile; ^3^Universidad San Sebastián,* *Facultad de Ciencias de la salud, Campus Los Leones, Santiago, Chile; ^4^Universidad Mayor sede Temuco, Av. Alemania 281, Temuco, Chile. ^5^University College London, Department of Primary Care and Population Health, Rowland Hill Street, London NW3 2PF, United Kingdom*

***** Author to whom correspondence should be addressed. Tel: +44 7 999070843; e-mail: christian.anfossi.19@ucl.ac.uk

**Data Summary**

| **Risk Factor** | **Outcome** | **Study** | **Title** | **Study design** | **Sex** | **Effect Measure** | **Overall Risk of Bias** |
| --- | --- | --- | --- | --- | --- | --- | --- |
| **Effort- Reward Imbalance** | Cerebrovascular disease | (Jood *et al.*, 2017) | The psychosocial work environment is associated with risk of stroke at working age | Case control study | Both | Odd Ratio: 1.28 (95% CI 1.01-1.62). P-value 0.04 | Low Risk of Bias |
| **Effort- Reward Imbalance** | Hypertensive disease | (Lamy *et al.*, 2014) | Psychosocial and organizational work factors and incidence of arterial hypertension among female healthcare workers: results of the Organisation des Soins et Sante des Soignants cohort | Cohort study | Females | Odd Ratio: Registered nurses 1.73 (95% CI 0.66 - 4.51); Nursing assistants 1.90 (95% CI 0.82 - 4.41). | Probably High Risk of Bias |
| **Effort- Reward Imbalance** | Hypertensive disease | (Gilbert-Ouimet *et al.*, 2012) | Repeated exposure to effort-reward imbalance, increased blood pressure, and hypertension incidence among white-collar workers Effort-reward imbalance and blood pressure | Cohort study | Both | Cumulative Incidence Ratio: Men 1.04 (0.56-1.95); Women <45 years old 1.28 (0.57-2.86) ≥45 years old 2.30 (1.16-4.55). | Low Risk of Bias |
| **Effort- Reward Imbalance** | Ischaemic heart disease | (Peter *et al.*, 2002) | Psychosocial work environment and myocardial infarction: improving risk estimation by combining two complementary job stress models in the SHEEP Study | Case control study | Both | Odd Ratio: Men 1.41 (1.05 - 1.89); Women 0.92 (0.53 - 1.61). | Probably Low Risk of Bias |
| **Effort- Reward Imbalance** | Ischaemic heart disease | (Kuper *et al.*, 2002) | When reciprocity fails: Effort-reward imbalance in relation to coronary heart disease and health functioning within the Whitehall II study | Cohort study | Both | Hazard Ratio: 1.36 (95% CI 1.12 to 1.65) | Probably Low Risk of Bias |
| **Effort- Reward Imbalance** | Ischaemic heart disease | (Bosma *et al.*, 1998) | Two alternative job stress models and the risk of coronary heart disease | Cohort study | Both | Odd Ratio: 2.52 (95%CI 1.36-4.65) | Low Risk of Bias |
| **Job Insecurity** | Cerebrovascular disease | (Slopen *et al.*, 2012) | Job strain, job insecurity, and incident cardiovascular disease in the women's health study: Results from a 10-year prospective study | Cohort study | Females | Hazard Ratio: 0.94 (95% CI 0.63 - 1.40) | Probably High Risk of Bias |
| **Job Insecurity** | Hypertensive disease | (Latza *et al.*, 2015) | Association of perceived job insecurity with ischemic heart disease and antihypertensive medication in the Danish Work Environment Cohort Study 1990-2010 | Cohort study | Both | Rate Ratio: 1.20 (95% CI 1.09-1.32) | Low Risk of Bias |
| **Job Insecurity** | Ischaemic heart disease | (Siegrist *et al.*, 1990) | Low status control, high effort at work and ischemic heart disease: Prospective evidence from blue-collar men | Cohort study | Males | Odd Ratio: 3.41 (95% CI 0.81-14.5) | Probably Low Risk of Bias |
| **Job Insecurity** | Ischaemic heart disease | (Netterstrøm *et al.*, 2010) | IS THE DEMAND-CONTROL MODEL STILL A USEFULL TOOL TO ASSESS WORK-RELATED PSYCHOSOCIAL RISK FOR ISCHEMIC HEART DISEASE? RESULTS FROM 14 YEAR FOLLOW UP IN THE COPENHAGEN CITY HEART STUDY | Cohort study | Both | Odd Ratio: Women 1.1 (0.3-4.3); Men 1.3 (0.5-3.3) | Probably Low Risk of Bias |
| **Job Insecurity** | Ischaemic heart disease | (Slopen *et al.*, 2012) | Job strain, job insecurity, and incident cardiovascular disease in the women's health study: Results from a 10-year prospective study | Cohort study | Females | Hazard Ratio: 1.35 (95% CI 0.95-1.92) | Probably High Risk of Bias |
| **Job Insecurity** | Ischaemic heart disease | (Ferrie *et al.*, 2013) | Job insecurity and incident coronary heart disease: The Whitehall II prospective cohort study | Cohort study | Both | Hazard Ratio: 1.42 (95% CI 1.05 - 1.93) | Low Risk of Bias |
| **Job Insecurity** | Ischaemic heart disease | (Latza *et al.*, 2015) | Association of perceived job insecurity with ischemic heart disease and antihypertensive medication in the Danish Work Environment Cohort Study 1990-2010 | Cohort study | Both | Rate Ratio: 1.20 (95% CI 0.95-1.52) | Low Risk of Bias |
| **Job Insecurity** | Ischaemic heart disease | (Cheng *et al.*, 2014) | Working hours, sleep duration and the risk of acute coronary heart disease: A case-control study of middle-aged men in Taiwan | Case control study | Males | Odd Ratio: Coronary heart disease 0.7 (95% CI 0.6 - 1.0), myocardial infarction 0.8 (95% CI 0.5 - 1.3) | Low Risk of Bias |
| **Job Strain** | Cerebrovascular disease | (Valery *et al.*, 2016) | Myocardial infarction and stroke: 16-year risk and stress at work in open population of 25-64-year-old women in Russia/Siberia (who Monica-psychosocial program) | Cohort study | Females | Hazard Ratio: 1.96 (95% CI 1.01-3.79) | Probably Low Risk of Bias |
| **Job Strain** | Cerebrovascular disease | (Tsutsumi, Kayaba and Ishikawa, 2011) | Impact of occupational stress on stroke across occupational classes and genders | Cohort study | Both | Hazard Ratio: Men 2.8 (1.2-6.4); Women 1.3 (0.6-3.0) | Probably Low Risk of Bias |
| **Job Strain** | Cerebrovascular disease | (Gafarov *et al.*, 2019) | Effect of stress at work on the risk of cardiovascular diseases among the population of 25-64 years in Russia/Siberia (WHO program "MONICA-psychosocial") | Cohort study | Both | Hazard Ratio: Men 3.8 (95% CI 1.6-8.8); Women 1.95 (95% CI 0.984-3.887) | Probably Low Risk of Bias |
| **Job Strain** | Cerebrovascular disease | (Schiöler *et al.*, 2015) | Psychosocial work environment and risk of ischemic stroke and coronary heart disease: a prospective longitudinal study of 75 236 construction workers | Cohort study | Males | Hazard Ratio: 1.10 (95% CI 0.79-1.53) | Probably High Risk of Bias |
| **Job Strain** | Cerebrovascular disease | (Slopen *et al.*, 2012) | Job strain, job insecurity, and incident cardiovascular disease in the women's health study: Results from a 10-year prospective study | Cohort study | Females | Hazard Ratio: 1.43 (95% CI 0.87 - 2.34) | Probably High Risk of Bias |
| **Job Strain** | Cerebrovascular disease | (Niedhammer *et al.*, 2020) | Psychosocial work exposures of the job strain model and cardiovascular mortality in France: results from the STRESSJEM prospective study. | Cohort study | Both | Cumulative exposure till 31/12/2002: Men 1.42 (95% CI 1.26-1.59), Women 1.39 (95% CI 1.18-1.63). | Probably High Risk of Bias |
| **Job Strain** | Cerebrovascular disease | (Tsutsumi *et al.*, 2009) | Prospective Study on Occupational Stress and Risk of Stroke | Cohort study | Both | Men 2.73 (1.17-6.38); Women 1.47 (0.63-3.40) | Low Risk of Bias |
| **Job Strain** | Cerebrovascular disease | (Jood *et al.*, 2017) | The psychosocial work environment is associated with risk of stroke at working age | Case control study | Both | Odd Ratio 1.30 (95% CI 1.05-1.62) | Low Risk of Bias |
| **Job Strain** | Hypertensive disease | (Radi *et al.*, 2005) | Job constraints and arterial hypertension: different effects in men and women: the IHPAF II case control study | Case control study | Both | Odd Ratio: Men 2.60 (1.15-5.85) Women 3.20 (0.92-11.12) | Probably High Risk of Bias |
| **Job Strain** | Hypertensive disease | (Schnall *et al.*, 1990) | The relationship between 'job strain,' workplace diastolic blood pressure, and left ventricular mass index. Results of a case-control study. | Case control study | Males | Odd Ratio: 3.09 (95% CI 1.30 - 7.30) | Low Risk of Bias |
| **Job Strain** | Hypertensive disease | (Huo Yung Kai *et al.*, 2018) | Impact of occupational environmental stressors on blood pressure changes and on incident cases of hypertension: a 5-year follow-up from the VISAT study | Cohort study | Both | Odd Ratio: Approximately 0.3 (95% CI 0.1-1.3) | High Risk of Bias |
| **Job Strain** | Ischaemic heart disease | (Orth-Gomér and Leineweber, 2005) | Multiple stressors and coronary disease in women. The Stockholm Female Coronary Risk Study. | Case control study | Females | Hazard Ratio: 4.5 (95% CI 2.5 - 8.4) | Probably Low Risk of Bias |
| **Job Strain** | Ischaemic heart disease | (Gafarov *et al.*, 2019) | Effect of stress at work on the risk of cardiovascular diseases among the population of 25-64 years in Russia/Siberia (WHO program "MONICA-psychosocial") | Cohort study | Both | Hazard Ratio: Men 1.15 (95% CI 0.6-2.2); Women 2.543 (95% CI 1.88-7.351) | Probably Low Risk of Bias |
| **Job Strain** | Ischaemic heart disease | (Kuper *et al.*, 2006) | Psychosocial determinants of coronary heart disease in middle-aged women: A prospective study in Sweden | Cohort study | Females | Hazard Ratio 1.4 (95% CI 0.7 - 2.7) | Probably Low Risk of Bias |
| **Job Strain** | Ischaemic heart disease | (Hallqvist *et al.*, 1998) | Is the effect of job strain on myocardial infarction risk due to interaction between high psychological demands and low decision latitude? Results from Stockholm Heart Epidemiology Program (SHEEP) | Case control study | Males | Relative Risk : Working men: Worst quartile vs the rest 2.2 (1.2 - 4.1) Cutoffs chosen to reflect an optimum balance between exposure contrast and power 9.2 (3.3 - 25.6) | Probably Low Risk of Bias |
| **Job Strain** | Ischaemic heart disease | (Hammar, Alfredsson and Johnson, 1998) | Job strain, social support at work, and incidence of myocardial infarction | Case control study | Both | Relative Risk: Men: 1.2 (95% CI 1.08 - 1.35); Women: 1.23 (95% CI 1.01 - 1.51); All subjects Age 30 - 54: 1.45 (95% CI 1.19 - 1.77) | Probably Low Risk of Bias |
| **Job Strain** | Ischaemic heart disease | (Netterstrøm *et al.*, 2010) | IS THE DEMAND-CONTROL MODEL STILL A USEFULL TOOL TO ASSESS WORK-RELATED PSYCHOSOCIAL RISK FOR ISCHEMIC HEART DISEASE? RESULTS FROM 14 YEAR FOLLOW UP IN THE COPENHAGEN CITY HEART STUDY | Cohort study | Both | Odd Ratio: Women 0.6 (0.1-3.4); Men 0.5 (0.1-1.0) | Probably Low Risk of Bias |
| **Job Strain** | Ischaemic heart disease | (Netterstrøm *et al.*, 1999) | Relation between job strain and myocardial infarction: a case-control study | Case control study | Males | Odd Ratio: 2.3 (95% CI 1.2 - 4.3) | Probably Low Risk of Bias |
| **Job Strain** | Ischaemic heart disease | (Wamala *et al.*, 2000) | Job stress and the occupational gradient in coronary heart disease risk in women - The Stockholm Female Coronary Risk Study | Case control study | Females | Odd Ratio: 2.32 (95% CI 1.21-4.45) | Probably Low Risk of Bias |
| **Job Strain** | Ischaemic heart disease | (Malinauskiene *et al.*, 2005) | Psychosocial factors at work and myocardial infarction among men in Kaunas, Lithuania | Case control study | Males | Odd Ratio: 0.73 (95% CI 0.38-1.39) | Probably Low Risk of Bias |
| **Job Strain** | Ischaemic heart disease | (Alterman *et al.*, 1994) | Decision latitude, psychologic demand, job strain, and coronary heart disease in the western electric study | Cohort study | Males | Relative Risk: 1.03 (95% CI 0.75-1.41) | Probably Low Risk of Bias |
| **Job Strain** | Ischaemic heart disease | (Kornitzer *et al.*, 2006) | Job stress and major coronary events: Results from the Job Stress, Absenteeism and Coronary Heart Disease in Europe study | Cohort study | Males | Hazard Ratio: 1.53 (95% CI 1.0 - 2.35) | Probably Low Risk of Bias |
| **Job Strain** | Ischaemic heart disease | (Peter *et al.*, 2002) | Psychosocial work environment and myocardial infarction: improving risk estimation by combining two complementary job stress models in the SHEEP Study | Case control study | Both | Odd Ratio: Men 1.39 (1.08 - 1.78); Women 1.68 (1.12 - 2.51). | Probably Low Risk of Bias |
| **Job Strain** | Ischaemic heart disease | (Ferrario *et al.*, 2017) | Job strain and the incidence of coronary heart diseases: does the association differ among occupational classes? A contribution from a pooled analysis of Northern Italian cohorts | Cohort study | Males | Hazard Ratio : Entire sample 1.57 (1.01-2.44) | Probably Low Risk of Bias |
| **Job Strain** | Ischaemic heart disease | (Valery *et al.*, 2016) | Myocardial infarction and stroke: 16-year risk and stress at work in open population of 25-64-year-old women in Russia/Siberia (who Monica-psychosocial program) | Cohort study | Females | Hazard Ratio: 3.22 (95% CI 1.15-9.04) | Probably Low Risk of Bias |
| **Job Strain** | Ischaemic heart disease | (Niedhammer *et al.*, 2020) | Psychosocial work exposures of the job strain model and cardiovascular mortality in France: results from the STRESSJEM prospective study. | Cohort study | Both | Hazard Ratio:Cumulative exposure till 31/12/2002: Men 1.22 (95% CI 1.13-1.32), Women 1.34 (95% CI 1.14-1.58). | Probably High Risk of Bias |
| **Job Strain** | Ischaemic heart disease | (Slopen *et al.*, 2012) | Job strain, job insecurity, and incident cardiovascular disease in the women's health study: Results from a 10-year prospective study | Cohort study | Females | Hazard Ratio: 1.67 (95% CI 1.04 - 2.70) | Probably High Risk of Bias |
| **Job Strain** | Ischaemic heart disease | (Schiöler *et al.*, 2015) | Psychosocial work environment and risk of ischemic stroke and coronary heart disease: a prospective longitudinal study of 75236 construction workers | Cohort study | Males | Hazard Ratio: 1.10 (95% CI 0.89-1.35) | Probably High Risk of Bias |
| **Job Strain** | Ischaemic heart disease | (Theorell *et al.*, 1998) | Decision latitude, job strain, and myocardial infarction: A study of working men in Stockholm | Case control study | Males | Odd Ratio: General 1.3 (1.0-1.8). Ages 45-54 1.8 (1.1 - 2.9); 55-64 1.0 (0.6 - 1.6). | Low Risk of Bias |
| **Job Strain** | Ischaemic heart disease | (Rugulies *et al.*, 2020) | Persistent and changing job strain and risk of coronary heart disease. A population-based cohort study of 1.6 million employees in Denmark. | Cohort study | Both | Hazard Ratio: Job strain and CHD (HR 1.10, 95% CI 1.07-1.13). Persistent job strain (HR 1.07, 95% CI 1.03-1.10), onset of job strain (HR 1.20, 95% CI 1.12-1.29) and removal of strain (HR 1.20, 95% CI 1.12-1.28) | Low Risk of Bias |
| **Job Strain** | Ischaemic heart disease | (Bobák *et al.*, 1998) | Association between psychosocial factors at work and nonfatal myocardial infarction in a population-based case-control study in Czech men | Case control study | Males | Odd Ratio: 1.31 (95% CI 0.77-2.25) | Low Risk of Bias |
| **Job Strain** | Ischaemic heart disease | (Ferrario *et al.*, 2019) | Exploring the interplay between job strain and different domains of physical activity on the incidence of coronary heart disease in adult men | Cohort study | Males | Hazard Ratio: 1.55 (95% CI 1.05-2.29) | Low Risk of Bias |
| **Job Strain** | Ischaemic heart disease | (Kivimäki *et al.*, 2006) | Why is evidence on job strain and coronary heart disease mixed? An illustration of measurement challenges in the Whitehall II Study | Cohort study | Both | Hazard Ratio: 1.30 (95% CI 1.13 - 1.51) | Low Risk of Bias |
| **Job Strain** | Ischaemic heart disease | (Kivimäki *et al.*, 2007) | Hypertension is not the link between job strain and coronary heart disease in the Whitehall II study | Cohort study | Both | Hazar Ratio: 1.36 (95% CI 1.07 - 1.72) | Low Risk of Bias |
| **Job Strain** | Ischaemic heart disease | (Mc Carthy, Perry and Greiner, 2012) | Age, job characteristics and coronary health | Case control study | Males | Odd Ratio: 0.56 (95% CI 0.13-2.51) | Low Risk of Bias |
| **Job Strain** | Ischaemic heart disease | (Netterstrým, Kristensen and Sjýl, 2006) | Psychological job demands increase the risk of ischaemic heart disease: A 14-year cohort study of employed Danish men | Cohort study | Males | Hazard Ratio: 2.6 (95% CI 1.1-6.1) | Low Risk of Bias |
| **Job Strain** | Ischaemic heart disease | (Kuper and Marmot, 2003) | Job strain, job demands, decision latitude, and risk of coronary heart disease within the Whitehall II study | Cohort study | Both | Hazard Ratio: 1.55 (95% CI 1.24 - 1.94) | Low Risk of Bias |
| **Job Strain** | Ischaemic heart disease | (De Bacquer *et al.*, 2005) | Perceived job stress and incidence of coronary events: 3-Year follow-up of the Belgian job stress project cohort | Cohort study | Males | Hazard Ratio: 1.38 (95% CI 0.80-2.38) | Low Risk of Bias |
| **Job Strain** | Ischaemic heart disease | (Bosma *et al.*, 1998) | Two alternative job stress models and the risk of coronary heart disease | Cohort study | Both | Odd Ratio: men 1.45 (1.03-2.06); women 1.14 (0.76-1.72) | Low Risk of Bias |
| **Job Strain** | Ischaemic heart disease | (Reed *et al.*, 1989) | Occupational strain and the incidence of coronary heart disease | Cohort study | Males | Hazar Ratio: 0.35 | High Risk of Bias |
| **Long Working Hours** | Cerebrovascular disease | (Hannerz *et al.*, 2018) | Long working hours and stroke among employees in the general workforce of Denmark. | Cohort study | Both | Rate Ratio: For overall stroke, 1.10 (95% CI 0.86–1.39) for 49–54 working hours and 0.89 (95% CI 0.69–1.16) for ≥55 working hours. Just for haemorrhagic stroke 49–54 weekly working hours had a rate ratio of 1.15 (95% CI 1.02–1.31) | Probably High Risk of Bias |
| **Long Working Hours** | Cerebrovascular disease | (Jeong *et al.*, 2013) | Working hours and cardiovascular disease in Korean workers: a case-control study. | Case control study | Both | Odd Ratio: Short-term working hours: 50.1−60 h SAH 1.19(0.49−2.89), ICH 1.73(0.81−3.69), CI 2.40(1.25−4.60). >60 h SAH 2.84(1.26−6.41), ICH 2.74(1.30−5.79), CI 6.10(3.33−11.2). Long-term working hours: 48.1−52 h SAH 1.43(0.54−3.76), ICH 1.88(0.65−5.49), CI 2.78(1.25−6.17) >52h 3.29 SAH 1.55(0.77−3.13), ICH 4.18(1.95−8.98), CI 5.17(2.78−9.63). | Probably High Risk of Bias |
| **Long Working Hours** | Cerebrovascular disease | (Hayashi *et al.*, 2019) | Working Hours and Risk of Acute Myocardial Infarction and Stroke Among Middle-Aged Japanese Men - The Japan Public Health Center-Based Prospective Study Cohort II | Cohort study | Males | Hazard Ratio: 0.83 (95% CI 0.60-1.13) | Low Risk of Bias |
| **Long Working Hours** | Cerebrovascular disease | (Alicandro *et al.*, 2020) | Long working hours and cardiovascular mortality: a census-based cohort study | Cohort study | Both | Hazard Radio: Men 49–54 H, HR 0.93 (95% CI 0.78–1.10), =>55 H 0.95 (95% CI 0.79–1.15). Women 49–54 H, HR 0.99 (95% CI0.67–1.46), =>55 H 0.98 (95% CI 0.62–1.53). | Low Risk of Bias |
| **Long Working Hours** | Hypertensive disease | (Nakanishi *et al.*, 1999) | Lifestyle and the development of hypertension: a 3-year follow-up study of middle-aged Japanese male office workers. | Cohort study | Males | Hazard Ratio: 0.58 (0.41-0.82) | High Risk of Bias |
| **Long Working Hours** | Hypertensive disease | (Hannerz, Larsen and Garde, 2018) | Long weekly working hours and ischaemic heart disease: a follow-up study among 145 861 randomly selected workers in Denmark | Cohort study | Both | Rate Ratio: 1.02 (95% CI 0.97 - 1.08) | High Risk of Bias |
| **Long Working Hours** | Ischaemic heart disease | (Kivimaki *et al.*, 2011) | Using additional information on working hours to predict coronary heart disease: a cohort study. | Cohort study | Both | Hazard Ratio: 1.67 (95% CI 1.10-2.55) | Probably Low Risk of Bias |
| **Long Working Hours** | Ischaemic heart disease | (Alfredsson, Karasek and Theorell, 1982) | Myocardial infarction risk and psychosocial work environment: an analysis of the male Swedish working force. | Case control study | Males | Relative Risk: 0.91 (95% CI 0.70-1.17) | Probably Low Risk of Bias |
| **Long Working Hours** | Ischaemic heart disease | (Hammar, Alfredsson and Theorell, 1994) | Job characteristics and the incidence of myocardial infarction | Case control study | Both | Relative Risk: Men 0.9 (95% CI 0.8-1.1); Women 1.0 (95% CI 0.7-1.5) | Probably Low Risk of Bias |
| **Long Working Hours** | Ischaemic heart disease | (Jeong *et al.*, 2013) | Working hours and cardiovascular disease in Korean workers: a case-control study. | Case control study | Both | Odd Ratio: Short-term working hours: 50.1−60 h, 2.11 (95% CI 1.03−4.33); >60 h 4.50 (2.23−9.08) Long-term working hours: 48.1−52 h 0.96 (95% CI 0.36−2.57); >52h 3.29 (95% CI 1.76−6.17) | Probably High Risk of Bias |
| **Long Working Hours** | Ischaemic heart disease | (Alicandro *et al.*, 2020) | Long working hours and cardiovascular mortality: a census-based cohort study | Cohort study | Both | Hazard Ratio: Men 49–54 H, HR 0.91 (95% CI 0.83–1.00), ≥55 H 0.95 (95% CI 0.86–1.05); Women 49–54 H, HR 0.92 (95% CI 0.61–1.37), ≥ 55 H 1.18 (95% CI 0.79–1.76). | Low Risk of Bias |
| **Long Working Hours** | Ischaemic heart disease | (Hayashi *et al.*, 2019) | Working Hours and Risk of Acute Myocardial Infarction and Stroke Among Middle-Aged Japanese Men - The Japan Public Health Center-Based Prospective Study Cohort II | Cohort study | Males | Hazard Ratio: General 1.63 (95% CI 1.01–2.63). Salaried employees (HR 2.11, 95% CI 1.03–4.35) and men aged 50–59 years (HR 2.60, 95% CI 1.42–4.77). | Low Risk of Bias |
| **Long Working Hours** | Ischaemic heart disease | (O’Reilly and Rosato, 2013) | Worked to death? A census-based longitudinal study of the relationship between the numbers of hours spent working and mortality risk | Cohort study | Both | Hazard Ratio: Men 49-54 hours/week 1.01 (0.91-1.12) Men 55 and over hours/week 0.96 (0.87-1.05) Women 49-54 hours/week 1.18 (0.88-1.58) Women 55 and over hours/week 0.86 (0.63-1.18) | Low Risk of Bias |
| **Long Working Hours** | Ischaemic heart disease | (Virtanen *et al.*, 2010) | Overtime work and incident coronary heart disease: the Whitehall II prospective cohort study | Cohort study | Both | Hazard Ratio: 1 h: 1.01 (0.76 - 1.34); 2 h: 1.28 (0.95 - 1.74); 3 - 4 h: 1.60 (1.15 -2.23) | Low Risk of Bias |
| **Long Working Hours** | Ischaemic heart disease | (Cheng *et al.*, 2014) | Working hours, sleep duration and the risk of acute coronary heart disease: A case-control study of middle-aged men in Taiwan | Case control study | Males | Odd Ratio: Coronary heart disease >48 - ≤60: 1.6 (95% CI 1.2 - 2.1), >60: 2.3 (95% CI 1.7 - 3.1). Myocardial infarction >48 - ≤60: 1.6 (95% CI 1.0 - 2.5), >60: 2.4 (95% CI 1.5 - 4.0). | Low Risk of Bias |
| **Long Working Hours** | Ischaemic heart disease | (Chen *et al.*, 2007) | Job categories and acute ischemic heart disease: A hospital-based, case-control study in Taiwan | Case control study | Both | Odd Ratio: 0.7 (95% CI 0.3 - 1.9) | High Risk of Bias |
| **Long Working Hours** | Ischaemic heart disease | (Hannerz, Larsen and Garde, 2018) | Long weekly working hours and ischaemic heart disease: a follow-up study among 145 861 randomly selected workers in Denmark | Cohort study | Both | Rate Ratio: 1.07 (95% CI 0.94 to 1.21) | High Risk of Bias |
| **Occupational Noise** | Cerebrovascular disease | (Eriksson *et al.*, 2018) | Longitudinal study of occupational noise exposure and joint effects with job strain and risk for coronary heart disease and stroke in Swedish men | Cohort study | Males | Hazard Ratio: 75-85 dB(A): 1.02 (0.85 to 1.22), >85dB(A): 1.16 (0.82 to 1.65) | Probably High Risk of Bias |
| **Occupational Noise** | Hypertensive disease | (Tong *et al.*, 2017) | Effect of Interaction Between Noise and A1166C Site of AT1R Gene Polymorphism on Essential Hypertension in an Iron and Steel Enterprise Workers | Case control study | Males | Odd Ratio: 1.726 (1.311-2.272) | Probably Low Risk of Bias |
| **Occupational Noise** | Hypertensive disease | (Lin *et al.*, 2020) | Relationship between time-varying exposure to occupational noise and incident hypertension: A prospective cohort study. | Cohort study | Both | Exposure 85-89 dBA-year:  Hazard Ratio: 1.36 (1.04-1.78) | Probably High Risk of Bias |
| **Occupational Noise** | Hypertensive disease | (Chang *et al.*, 2013) | Occupational Noise Exposure and Incident Hypertension in Men: A Prospective Cohort Study | Cohort study | Males | Relative Risk: 1.93 (95% CI 1.15 - 3.22) | Probably High Risk of Bias |
| **Occupational Noise** | Ischaemic heart disease | (Virkkunen, Kauppinen and Tenkanen, 2005) | Long-term effect of occupational noise on the risk of coronary heart disease | Cohort study | Males | Relative Risk: - Continuous: 80-85 dB: 1.32 (1.16-1.5); >85 bB 1.48 (1.28-1.71). -Impulse: 1.34 (1.12-1.60) - Both adjusted 1.46 (1.21-1.76) | Probably Low Risk of Bias |
| **Occupational Noise** | Ischaemic heart disease | (Eriksson *et al.*, 2018) | Longitudinal study of occupational noise exposure and joint effects with job strain and risk for coronary heart disease and stroke in Swedish men | Cohort study | Males | Hazard Ratio: 75-85 dB(A): 1.15 (95% CI 1.01 - 1.31). >85dB(A) 1.27 (95% CI 0.99 - 1.63). | Probably High Risk of Bias |
| **Occupational Noise** | Ischaemic heart disease | (Davies *et al.*, 2005) | Occupational exposure to noise and mortality from acute myocardial infarction | Cohort study | No reported | Relative Risk: Thresholds > 85 dB(A) > 3 years of exposure: 1.1 (95% CI 0.94 - 1.3) Thresholds > 95 dB(A) > 19 years of exposure: 1.5 (95% CI 1.1 - 2.2) | Probably High Risk of Bias |
| **Shift Work** | Cerebrovascular disease | (Virtanen and Notkol, 2002) | Socioeconomic inequalities in cardiovascular mortality and the role of work: a register study of Finnish men | Cohort study | Males | Rate Rario: Two-shift, evening 1.19 (1.01-1.39); Three-shift, night 1.06 (0.86-1.31) | Probably Low Risk of Bias |
| **Shift Work** | Cerebrovascular disease | (Fujino *et al.*, 2006) | A prospective cohort study of shift work and risk of ischemic heart disease in Japanese male workers. | Cohort study | Males | Relative Risk: 1.12 (95% CI 0.66 - 1.91) | Probably Low Risk of Bias |
| **Shift Work** | Cerebrovascular disease | (Jørgensen *et al.*, 2017) | Shift work and overall and cause-specific mortality in the Danish nurse cohort | Cohort study | Females | Hazard Ratio: Evening shifts 1.00 (0.45-2.23) Night shifts 2.27 (1.10-4.67) Rotating shifts 1.20 (0.68-2.14) | Probably High Risk of Bias |
| **Shift Work** | Cerebrovascular disease | (Fujino, 2007) | Occupational factors and mortality in the Japan Collaborative Cohort Study for Evaluation of Cancer (JACC). | Cohort study | Both | Hazard Ratio:  MALES: -Mainly night: 1.08 (0.81, 1.45) -Alternate: 1.19 (0.95, 1.50)  FEMALES: -Mainly night: 1.08 (0.90, 1.30) -Alternate: 1.03 (0.83, 1.29) | Probably High Risk of Bias |
| **Shift Work** | Cerebrovascular disease | (Brown *et al.*, 2009) | Rotating night shift work is associated with ischemic stroke risk | Cohort study | Females | Hazard Ratio: For every 5 years, 1.06 (95% CI 1.02 - 1.10) | Low Risk of Bias |
| **Shift Work** | Cerebrovascular disease | (Gu *et al.*, 2015) | Total and Cause-Specific Mortality of US Nurses Working Rotating Night Shifts | Cohort study | Females | Hazard Ratio: 1-5 years 1.02 (0.87, 1.20), 6-14 years 1.20 (CI 95% 0.97 - 1.48); ≥15 years 1.12 (CI 95% 0.88 - 1.42) | Low Risk of Bias |
| **Shift Work** | Hypertensive disease | (Itani *et al.*, 2017) | Short sleep duration, shift work, and actual days taken off work are predictive life-style risk factors for new-onset metabolic syndrome: a seven-year cohort study of 40,000 male workers. | Cohort study | Males | Hazard Ratio: 1.06 (1.01-1.10) | Probably Low Risk of Bias |
| **Shift Work** | Hypertensive disease | (Biggi *et al.*, 2008) | Metabolic syndrome in permanent night workers | Cohort study | Males | Odd Ratio: 0.9 (0.6 - 1.2) | Probably Low Risk of Bias |
| **Shift Work** | Hypertensive disease | (Li *et al.*, 2017) | Prospective Cohort Study to Elucidate the Correlation between Occupational Stress and Hypertension Risk in Oil Workers from Kelamayi City in the Xinjiang Uygur Autonomous Region of China | Cohort study | Both | Hazard Ratio: 1.131 (CI 95% 0.840-1.522) | Probably Low Risk of Bias |
| **Shift Work** | Hypertensive disease | (Morikawa *et al.*, 1999) | Relationship between shift work and onset of hypertension in a cohort of manual workers | Cohort study | Males | Relative Risk: 18-29 years, Sift-shift 4.0 (95% CI 1.67-9.67);  40-49 years, Sift-day 2.5 (95% CI 1.08-5.91) | Probably Low Risk of Bias |
| **Shift Work** | Hypertensive disease | (Zayeri, Amini and Hasanzadeh, 2018) | Assessment of relationship between shift work and hypertension in Mahshahr petrochemical Ataff: A longitudinal study | Cohort study | Both | Odd Ratio: 1.04 (95% CI 0.98 - 1.10) | Probably High Risk of Bias |
| **Shift Work** | Hypertensive disease | (Guo *et al.*, 2017) | [Research on potential interaction between mitochondrial DNA copy number and related factors on risk of hypertension in coal miners]. | Case control study | Both | Odd Ratio: 0.69 (0.48-0.99) | Probably High Risk of Bias |
| **Shift Work** | Hypertensive disease | (Kubo *et al.*, 2013) | An Industry-Based Cohort Study of the Association Between Weight Gain and Hypertension Risk Among Rotating Shift Workers | Cohort study | Males | Hazard Ratio: 1.88 (95% CI 1.71-2.07) | Probably High Risk of Bias |
| **Shift Work** | Hypertensive disease | (Hublin *et al.*, 2010) | Shift-work and cardiovascular disease: a population-based 22-year follow-up study | Cohort study | Both | Hazard Ratio:  Night-time work either in 1975 or 1981: Men: 0.72 (0.37 - 1.39); Women: 1.02 (0.70 - 1.48). Shift-work both in 1975 and 1981: Men: 1.07 (0.88 - 1.30); Women: 1.00 (0.80 - 1.23). | Probably High Risk of Bias |
| **Shift Work** | Hypertensive disease | (Jørgensen *et al.*, 2017) | Shift work and overall and cause-specific mortality in the Danish nurse cohort | Cohort study | Females | Hazard Ratio:  Evening shifts 1.57 (0.59-4.22) Night shifts 3.30 (1.32-8.28) Rotating shifts 1.68 (0.78-3.62) | Probably High Risk of Bias |
| **Shift Work** | Hypertensive disease | (Hulsegge *et al.*, 2019) | Shift work, chronotype and the risk of cardiometabolic risk factors | Cohort study | Both | Odd Ratio: 0.99 (95% CI 0.68 - 1.43) | Low Risk of Bias |
| **Shift Work** | Hypertensive disease | (Larsen *et al.*, 2020) | Night work and the risk of ischemic heart disease and anti-hypertensive drug use. A cohort study of 145 861 Danish employees | Cohort study | Both | Relative Risk: 1.05 (95% CI 1.01-1.09) | Low Risk of Bias |
| **Shift Work** | Hypertensive disease | (Ferguson *et al.*, 2019) | Night and rotational work exposure within the last 12 months and risk of incident hypertension | Cohort study | Both | Hazard Ratio: Any night shifts HR: 2.27 (95% CI 1.08-4.80) Mostly non-night work & frequent rotations HR: 2.39 (95% CI 1.10-5.20) Mostly night work & frequent rotations HR: 4.00 (CI 95% 1.69-9.52) | High Risk of Bias |
| **Shift Work** | Ischaemic heart disease | (Fujino *et al.*, 2006) | A prospective cohort study of shift work and risk of ischemic heart disease in Japanese male workers. | Cohort study | Males | Relative Risk: 2.32 (95% CI 1.37 - 3.95) | Probably Low Risk of Bias |
| **Shift Work** | Ischaemic heart disease | (Vetter *et al.*, 2016) | Association Between Rotating Night Shift Work and Risk of Coronary Heart Disease Among Women | Cohort study | Females | Hazard Ratio: Women Without Diabetes, Hypertension, or Hypercholesterolemia. NHS: 5-9y 1.37 (1.15-1.63); ≥10y 1.36 (1.17-1.57). NHS2: 5-9y 1.17 (1.00 -1.38); ≥10y 1.28 (1.06-1.54). | Probably Low Risk of Bias |
| **Shift Work** | Ischaemic heart disease | (Tenkanen *et al.*, 1997) | Shift work, occupation and coronary heart disease over 6 years of follow-up in the Helsinki Heart Study | Cohort study | Males | Relative Risk: 1.52 (1.11 - 2.07) | Probably Low Risk of Bias |
| **Shift Work** | Ischaemic heart disease | (Virkkunen *et al.*, 2006) | The triad of shift work, occupational noise, and physical workload and risk of coronary heart disease | Cohort study | Males | Relative Risk: 1.37 (95% CI 1.11-1.70) | Probably Low Risk of Bias |
| **Shift Work** | Ischaemic heart disease | (Biggi *et al.*, 2008) | Metabolic syndrome in permanent night workers | Cohort study | Males | Risk Ratio: 2.0 (95% CI 0.4-9.4) | Probably Low Risk of Bias |
| **Shift Work** | Ischaemic heart disease | (Alfredsson, Karasek and Theorell, 1982) | Myocardial infarction risk and psychosocial work environment: an analysis of the male Swedish working force. | Case control study | Males | Relative Risk: 1.25 (95% CI 0.97-1.62) | Probably Low Risk of Bias |
| **Shift Work** | Ischaemic heart disease | (Fujino, 2007) | Occupational factors and mortality in the Japan Collaborative Cohort Study for Evaluation of Cancer (JACC). | Cohort study | Both | Hazard Ratio:  MALES: -Mainly night: 1.07 (0.69-1.67) -Alternate: 1.76 (1.34-2.33)  FEMALES: -Mainly night: 0.97 (0.48-1.97) -Alternate: 1.10 (0.69-1.73) | Probably High Risk of Bias |
| **Shift Work** | Ischaemic heart disease | (Kleiven, Bøggild and Jeppesen, 1998) | Shift work and sick leave | Case control study | Both | Odd Ratio: 0.75 (0.42-1.31) | Probably High Risk of Bias |
| **Shift Work** | Ischaemic heart disease | (Tochsen, 1993) | Working hours and ischaemic heart disease in Danish men: a 4-year cohort study of hospitalization. | Cohort study | Males | Standardized hospitalization ratios (SHR): Night/early morning work 193 (90% CI 158.3-236.0); Late evening work 215 (90% CI 192.4-240.1); Rosters covering 24-hour services 168 (90% CI 151.8-185.5); Other irregular working hours 172 (90% CI 166.4-182.1) | Probably High Risk of Bias |
| **Shift Work** | Ischaemic heart disease | (Bøggild *et al.*, 1999) | Shift work, social class and ischemic heart disease in middle-aged and elderly men. A 22-year follow-up in the "Copenhagen Male Study". | Cohort study | Males | Relative Risk: 1.0 (95% CI 0.9-1.2) | Probably High Risk of Bias |
| **Shift Work** | Ischaemic heart disease | (Hublin *et al.*, 2010) | Shift-work and cardiovascular disease: a population-based 22-year follow-up study | Cohort study | Both | Hazard Ratio: Night-time work either in 1975 or 1981:  Men: 1.82 (95% CI 0.97-3.41); Women: 0.90 (95% CI 0.36-2.23). Shift-work both in 1975 and 1981:  Men: 1.06 (95% CI 0.75-1.50); Women: 1.21 (95% CI 0.75-1.93). | Probably High Risk of Bias |
| **Shift Work** | Ischaemic heart disease | (Jørgensen *et al.*, 2017) | Shift work and overall and cause-specific mortality in the Danish nurse cohort | Cohort study | Females | Hazard Ratio:  Evening shifts 2.30 (1.15-4.60) Night shifts 4.10 (2.05-8.22) Rotating shifts 1.28 (0.65-2.21) | Probably High Risk of Bias |
| **Shift Work** | Ischaemic heart disease | (Larsen *et al.*, 2020) | Night work and the risk of ischemic heart disease and anti-hypertensive drug use. A cohort study of 145 861 Danish employees | Cohort study | Both | Relative Risk: 1.08 (95% CI 0.98-1.19) | Low Risk of Bias |
| **Shift Work** | Ischaemic heart disease | (Gu *et al.*, 2015) | Total and Cause-Specific Mortality of US Nurses Working Rotating Night Shifts | Cohort study | Females | Hazard Ratio: 1-5 years: 0.97 (0.85-1.12), 6-14 years: 1.23 (1.03-1.47), 15 years ≥ 1.34 (1.11-1.61) | Low Risk of Bias |
| **Shift Work** | Ischaemic heart disease | (Knutsson *et al.*, 1999) | Shiftwork and myocardial infarction: a case-control study | Case control study | Both | Odd Ratio: Men 1.3 (95% CI 1.1 - 1.6); Women 1.3 (95% CI 0.9 - 1.8) | Low Risk of Bias |
| **Shift Work** | Ischaemic heart disease | (Wang *et al.*, 2016) | Shift work and 20-year incidence of acute myocardial infarction: results from the Kuopio Ischemic Heart Disease Risk Factor Study | Cohort study | Males | Hazarrd Ratio: Men without IHD (N=1565) at baseline 1.09 (0.87-1.37) | Low Risk of Bias |
| **Shift Work** | Ischaemic heart disease | (Cheng *et al.*, 2014) | Working hours, sleep duration and the risk of acute coronary heart disease: A case-control study of middle-aged men in Taiwan | Case control study | Males | Odd Ratio: Coronary heart disease 0.9 (95% CI 0.7 - 1.2), myocardial infarction 0.7 (95% CI 0.4 - 1.1) | Low Risk of Bias |
| **Shift Work** | Ischaemic heart disease | (Yong *et al.*, 2014) | Shift work and risk of non-cancer mortality in a cohort of German male chemical workers. | Cohort study | Males | Hazard Ratio: 0.77 (95% CI 0.52-1.14) | High Risk of Bias |

**References:**

Alfredsson, L., Karasek, R. and Theorell, T. (1982) ‘Myocardial infarction risk and psychosocial work environment: An analysis of the male Swedish working force’, *Social Science and Medicine*, 16(4), pp. 463–467. doi: 10.1016/0277-9536(82)90054-5.

Alicandro, G. *et al.* (2020) ‘Long working hours and cardiovascular mortality: a census-based cohort study’, *International Journal of Public Health*. Springer International Publishing, 65(3), pp. 257–266. doi: 10.1007/s00038-020-01361-y.

Alterman, T. *et al.* (1994) ‘Decision latitude, psychologic demand, job strain, and coronary heart disease in the western electric study’, *American Journal of Epidemiology*, 139(6), pp. 620–627. doi: 10.1093/oxfordjournals.aje.a117051.

De Bacquer, D. *et al.* (2005) ‘Perceived job stress and incidence of coronary events: 3-Year follow-up of the Belgian job stress project cohort’, *American Journal of Epidemiology*, 161(5), pp. 434–441. doi: 10.1093/aje/kwi040.

Biggi, N. *et al.* (2008) ‘Metabolic syndrome in permanent night workers’, *Chronobiology International*, 25(2–3), pp. 443–454. doi: 10.1080/07420520802114193.

Bobák, M. *et al.* (1998) ‘Association between psychosocial factors at work and nonfatal myocardial infarction in a population-based case-control study in Czech men’, *Epidemiology*. M. Bobak, Dept. of Epidemiology/Public Health, University College London, 1-19 Torrington Place, London WC1E 6BT, United Kingdom: Lippincott Williams and Wilkins (351 West Camden Street, Baltimore MD 21201-2436, United States), 9(1), pp. 43–47. doi: http://dx.doi.org/10.1097/00001648-199801000-00010.

Bøggild, H. *et al.* (1999) ‘Shift work, social class, and ischaemic heart disease in middle aged and elderly men; A 22 year follow up in the Copenhagen male study’, *Occupational and Environmental Medicine*, 56(9), pp. 640–645. doi: 10.1136/oem.56.9.640.

Bosma, H. *et al.* (1998) ‘Two alternative job stress models and the risk of coronary heart disease’, *American Journal of Public Health*, 88(1), pp. 68–74. doi: 10.2105/AJPH.88.1.68.

Brown, D. L. *et al.* (2009) ‘Rotating night shift work and the sisk of ischemic stroke’, *American Journal of Epidemiology*, 169(11), pp. 1370–1377. doi: 10.1093/aje/kwp056.

Chang, T. Y. *et al.* (2013) ‘Occupational noise exposure and incident hypertension in men: A prospective cohort study’, *American Journal of Epidemiology*, 177(8), pp. 818–825. doi: 10.1093/aje/kws300.

Chen, J. D. *et al.* (2007) ‘Job categories and acute ischemic heart disease: A hospital-based, case-control study in Taiwan’, *American Journal of Industrial Medicine*, 50(6), pp. 409–414. doi: 10.1002/ajim.20462.

Cheng, Y. *et al.* (2014) ‘Working hours, sleep duration and the risk of acute coronary heart disease: A case-control study of middle-aged men in Taiwan’, *International Journal of Cardiology*. Elsevier Ireland Ltd, 171(3), pp. 419–422. doi: 10.1016/j.ijcard.2013.12.035.

Davies, H. W. *et al.* (2005) ‘Occupational exposure to noise and mortality from acute myocardial infarction’, *Epidemiology*, 16(1), pp. 25–32. doi: 10.1097/01.ede.0000147121.13399.bf.

Eriksson, H. P. *et al.* (2018) ‘Longitudinal study of occupational noise exposure and joint effects with job strain and risk for coronary heart disease and stroke in Swedish men’, *BMJ Open*, 8(4). doi: 10.1136/bmjopen-2017-019160.

Ferguson, J. M. *et al.* (2019) ‘Night and rotational work exposure within the last 12 months and risk of incident hypertension’, *Scandinavian Journal of Work, Environment and Health*, 45(3), pp. 256–266. doi: 10.5271/sjweh.3788.

Ferrario, M. M. *et al.* (2017) ‘Job strain and the incidence of coronary heart diseases: Does the association differ among occupational classes? A contribution from a pooled analysis of Northern Italian cohorts’, *BMJ Open*, 7(1), pp. 1–8. doi: 10.1136/bmjopen-2016-014119.

Ferrario, M. M. *et al.* (2019) ‘Exploring the interplay between job strain and different domains of physical activity on the incidence of coronary heart disease in adult men’, *European Journal of Preventive Cardiology*, 26(17), pp. 1877–1885. doi: 10.1177/2047487319852186.

Ferrie, J. E. *et al.* (2013) ‘Job insecurity and incident coronary heart disease: The Whitehall II prospective cohort study’, *Atherosclerosis*. Elsevier Ltd, 227(1), pp. 178–181. doi: 10.1016/j.atherosclerosis.2012.12.027.

Fujino, Y. *et al.* (2006) ‘A prospective cohort study of shift work and risk of ischemic heart disease in Japanese male workers’, *American Journal of Epidemiology*, 164(2), pp. 128–135. doi: 10.1093/aje/kwj185.

Fujino, Y. (2007) ‘Occupational factors and mortality in the Japan Collaborative Cohort Study for Evaluation of Cancer (JACC).’, *Asian Pacific journal of cancer prevention : APJCP*, 8 Suppl(May), pp. 97–104.

Gafarov, V. V. *et al.* (2019) ‘Effect of stress at work on the risk of cardiovascular diseases among the population of 25-64 years in Russia/Siberia (WHO program “MONICA-psychosocial”)’, *Terapevticheskii Arkhiv*, 91(2), pp. 13–18. doi: 10.26442/00403660.2019.01.000022.

Gilbert-Ouimet, M. *et al.* (2012) ‘Repeated exposure to effort-reward imbalance, increased blood pressure, and hypertension incidence among white-collar workers. Effort-reward imbalance and blood pressure’, *Journal of Psychosomatic Research*. Elsevier Inc., 72(1), pp. 26–32. doi: 10.1016/j.jpsychores.2011.07.002.

Gu, F. *et al.* (2015) ‘Total and cause-specific mortality of U.S. nurses working rotating night shifts’, *American Journal of Preventive Medicine*. Elsevier, 48(3), pp. 241–252. doi: 10.1016/j.amepre.2014.10.018.

Guo, J. Y. *et al.* (2017) ‘[Research on potential interaction between mitochondrial DNA copy number and related factors on risk of hypertension in coal miners].’, *Zhonghua liu xing bing xue za zhi = Zhonghua liuxingbingxue zazhi*. China, 38(1), pp. 26–31. doi: 10.3760/cma.j.issn.0254-6450.2017.01.005.

Hallqvist, J. *et al.* (1998) ‘Is the effect of job strain on myocardial infarction risk due to interaction between high psychological demands and low decision latitude? Results from Stockholm Heart Epidemiology Program (SHEEP)’, *Social Science and Medicine*, 46(11), pp. 1405–1415. doi: 10.1016/S0277-9536(97)10084-3.

Hammar, N., Alfredsson, L. and Johnson, J. V. (1998) ‘Job strain, social support at work, and incidence of myocardial infarction’, *Occupational and Environmental Medicine*, 55(8), pp. 548–553. doi: 10.1136/oem.55.8.548.

Hammar, N., Alfredsson, L. and Theorell, T. (1994) ‘Job characteristics and the incidence of myocardial infarction’, *International Journal of Epidemiology*, 23(2), pp. 277–284. doi: 10.1093/ije/23.2.277.

Hannerz, H. *et al.* (2018) ‘Long working hours and stroke among employees in the general workforce of Denmark’, *Scandinavian Journal of Public Health*, 46(3), pp. 368–374. doi: 10.1177/1403494817748264.

Hannerz, H., Larsen, A. D. and Garde, A. H. (2018) ‘Long weekly working hours and ischaemic heart disease: A follow-up study among 145 861 randomly selected workers in Denmark’, *BMJ Open*, 8(6). doi: 10.1136/bmjopen-2017-019807.

Hayashi, R. *et al.* (2019) ‘Working hours and risk of acute myocardial infarction and stroke among middle-aged japanese men: The Japan public health center-based prospective study cohort II’, *Circulation Journal*, 83(5), pp. 1072–1079. doi: 10.1253/circj.CJ-18-0842.

Hublin, C. *et al.* (2010) ‘Shift-work and cardiovascular disease: A population-based 22-year follow-up study’, *European Journal of Epidemiology*, 25(5), pp. 315–323. doi: 10.1007/s10654-010-9439-3.

Hulsegge, G. *et al.* (2019) ‘Shift work, chronotype and the risk of cardiometabolic risk factors’, *European Journal of Public Health*, 29(1), pp. 128–134. doi: 10.1093/eurpub/cky092.

Huo Yung Kai, S. *et al.* (2018) ‘Impact of occupational environmental stressors on blood pressure changes and on incident cases of hypertension: A 5-year follow-up from the VISAT study’, *Environmental Health: A Global Access Science Source*. Environmental Health, 17(1), pp. 1–10. doi: 10.1186/s12940-018-0423-9.

Itani, O. *et al.* (2017) ‘Short sleep duration, shift work, and actual days taken off work are predictive life-style risk factors for new-onset metabolic syndrome: a seven-year cohort study of 40,000 male workers’, *Sleep Medicine*. Elsevier Ltd, 39, pp. 87–94. doi: 10.1016/j.sleep.2017.07.027.

Jeong, I. *et al.* (2013) ‘Working hours and cardiovascular disease in Korean workers: A case-control study’, *Journal of Occupational Health*, 55(5), pp. 385–391. doi: 10.1539/joh.12-0245-OA.

Jood, K. *et al.* (2017) ‘The psychosocial work environment is associated with risk of stroke at working age’, *Scandinavian Journal of Work, Environment and Health*, 43(4), pp. 367–374. doi: 10.5271/sjweh.3636.

Jørgensen, J. T. *et al.* (2017) ‘Shift work and overall and cause-specific mortality in the Danish nurse cohort’, *Scandinavian Journal of Work, Environment and Health*, 43(2), pp. 117–126. doi: 10.5271/sjweh.3612.

Kivimaki, M. *et al.* (2011) ‘Using additional information on working hours to predict coronary heart disease’, *Annals of Internal Medicine*. M. Kivimaki, Department of Epidemiology and Public Health, University College London, 1-19 Torrington Place, London WC1E 6BT, United Kingdom. E-mail: m.kivimaki@ucl.ac.uk: American College of Physicians (190 N. Indenpence Mall West, Philadelphia PA 19106-1572, United States), 154(7), pp. 457–463. doi: http://dx.doi.org/10.7326/0003-4819-154-7-201104050-00003.

Kivimäki, M. *et al.* (2006) ‘Why is evidence on job strain and coronary heart disease mixed? An illustration of measurement challenges in the Whitehall II study’, *Psychosomatic Medicine*, 68(3), pp. 398–401. doi: 10.1097/01.psy.0000221252.84351.e2.

Kivimäki, M. *et al.* (2007) ‘Hypertension Is Not the Link Between Job Strain and Coronary Heart Disease in the Whitehall II Study’, *American Journal of Hypertension*, 20(11), pp. 1146–1153. doi: 10.1016/j.amjhyper.2007.06.006.

Kleiven, M., Bøggild, H. and Jeppesen, H. J. (1998) ‘Shift work and sick leave’, *Scandinavian Journal of Work, Environment and Health*, 24(SUPPL. 3), pp. 128–133.

Knutsson, A. *et al.* (1999) ‘Shiftwork and myocardial infarction: A case-control study’, *Occupational and Environmental Medicine*, 56(1), pp. 46–50. doi: 10.1136/oem.56.1.46.

Kornitzer, M. *et al.* (2006) ‘Job stress and major coronary events: Results from the Job Stress, Absenteeism and Coronary Heart Disease in Europe study’, *European Journal of Preventive Cardiology*, 13(5), pp. 695–704. doi: 10.1097/01.hjr.0000221865.19415.e9.

Kubo, T. *et al.* (2013) ‘An industry-based cohort study of the association between weight gain and hypertension risk among rotating shift workers’, *Journal of Occupational and Environmental Medicine*, 55(9), pp. 1041–1045. doi: 10.1097/JOM.0b013e31829731fd.

Kuper, H. *et al.* (2002) ‘When reciprocity fails: Effort-reward imbalance in relation to coronary heart disease and health functioning within the Whitehall II study’, *Occupational and Environmental Medicine*, 59(11), pp. 777–784. doi: 10.1136/oem.59.11.777.

Kuper, H. *et al.* (2006) ‘Psychosocial determinants of coronary heart disease in middle-aged women: A prospective study in Sweden’, *American Journal of Epidemiology*, 164(4), pp. 349–357. doi: 10.1093/aje/kwj212.

Kuper, H. and Marmot, M. (2003) ‘Job strain, job demands, decision latitude, and risk of coronary heart disease within the Whitehall II study’, *Journal of Epidemiology and Community Health*, 57(2), pp. 147–153. doi: 10.1136/jech.57.2.147.

Lamy, S. *et al.* (2014) ‘Psychosocial and organizational work factors and incidence of arterial hypertension among female healthcare workers: Results of the Organisation des Soins et Santé des Soignants cohort’, *Journal of Hypertension*, 32(6), pp. 1229–1236. doi: 10.1097/HJH.0000000000000155.

Larsen, A. D. *et al.* (2020) ‘Night work and risk of ischaemic heart disease and anti-hypertensive drug use: A cohort study of 145 861 Danish employees’, *European Journal of Public Health*, 30(2), pp. 259–264. doi: 10.1093/EURPUB/CKZ189.

Latza, U. *et al.* (2015) ‘Association of perceived job insecurity with ischemic heart disease and antihypertensive medication in the Danish Work Environment Cohort Study 1990–2010’, *International Archives of Occupational and Environmental Health*. Springer Berlin Heidelberg, 88(8), pp. 1087–1097. doi: 10.1007/s00420-015-1030-5.

Li, R. *et al.* (2017) ‘Prospective cohort study to elucidate the correlation between occupational stress and hypertension risk in oil workers from kelamayi city in the xinjiang uygur autonomous region of China’, *International Journal of Environmental Research and Public Health*, 14(1). doi: 10.3390/ijerph14010001.

Lin, Y. T. *et al.* (2020) ‘Relationship between time-varying exposure to occupational noise and incident hypertension: A prospective cohort study’, *International Journal of Hygiene and Environmental Health*. Elsevier, 226(1018), p. 113487. doi: 10.1016/j.ijheh.2020.113487.

Malinauskiene, V. *et al.* (2005) ‘Psychosocial factors at work and myocardial infarction among men in Kaunas, Lithuania’, *Scandinavian Journal of Work, Environment and Health*, 31(3), pp. 218–223. doi: 10.5271/sjweh.872.

Mc Carthy, V. J. C., Perry, I. J. and Greiner, B. A. (2012) ‘Age, job characteristics and coronary health’, *Occupational Medicine*, 62(8), pp. 613–619. doi: 10.1093/occmed/kqs139.

Morikawa, Y. *et al.* (1999) ‘Relationship between shift work and onset of hypertension in a cohort of manual workers’, *Scandinavian Journal of Work, Environment and Health*, 25(2), pp. 100–104. doi: 10.5271/sjweh.411.

Nakanishi, N. *et al.* (1999) ‘Lifestyle and the development of hypertension: A 3-year follow-up study of middle-aged Japanese male office workers’, *Occupational Medicine*, 49(2), pp. 109–114. doi: 10.1093/occmed/49.2.109.

Netterstrøm, B. *et al.* (1999) ‘Relation between job strain and myocardial infarction: A case-control study’, *Occupational and Environmental Medicine*, 56(5), pp. 339–342. doi: 10.1136/oem.56.5.339.

Netterstrøm, B. *et al.* (2010) ‘Is the demand-control model still a usefull tool to assess work-related psychosocial risk for ischemic heart disease? Results from 14 year follow up in the Copenhagen City Heart study’, *International Journal of Occupational Medicine and Environmental Health*, 23(3), pp. 217–224. doi: 10.2478/v10001-010-0031-6.

Netterstrým, B., Kristensen, T. S. and Sjýl, A. (2006) ‘Psychological job demands increase the risk of ischaemic heart disease: A 14-year cohort study of employed Danish men’, *European Journal of Preventive Cardiology*, 13(3), pp. 414–420. doi: 10.1097/01.hjr.0000201512.05720.87.

Niedhammer, I. *et al.* (2020) ‘Psychosocial work exposures of the job strain model and cardiovascular mortality in France: Results from the STRESSJEM prospective study’, *Scandinavian Journal of Work, Environment and Health*, 46(5), pp. 542–551. doi: 10.5271/sjweh.3902.

O’Reilly, D. and Rosato, M. (2013) ‘Worked to death? A census-based longitudinal study of the relationship between the numbers of hours spent working and mortality risk’, *International Journal of Epidemiology*, 42(6), pp. 1820–1830. doi: 10.1093/ije/dyt211.

Orth-Gomér, K. and Leineweber, C. (2005) ‘Multiple stressors and coronary disease in women: The Stockholm Female Coronary Risk Study’, *Biological Psychology*, 69(1 SPEC. ISS.), pp. 57–66. doi: 10.1016/j.biopsycho.2004.11.005.

Peter, R. *et al.* (2002) ‘Psychosocial work environment and myocardial infarction: Improving risk estimation by combining two complementary job stress models in the SHEEP study’, *Journal of Epidemiology and Community Health*, 56(4), pp. 294–300. doi: 10.1136/jech.56.4.294.

Radi, S. *et al.* (2005) ‘Job constraints and arterial hypertension: Different effects in men and women: The IHPAF II case control study’, *Occupational and Environmental Medicine*, 62(10), pp. 711–717. doi: 10.1136/oem.2004.012955.

Reed, D. M. *et al.* (1989) ‘Occupational strain and the incidence of coronary heart disease’, *American Journal of Epidemiology*, 129(3), pp. 495–502. doi: 10.1093/oxfordjournals.aje.a115160.

Rugulies, R. *et al.* (2020) ‘Persistent and changing job strain and risk of coronary heart disease. A population-based cohort study of 1.6 million employees in Denmark’, *Scandinavian Journal of Work, Environment and Health*, 46(5), pp. 498–507. doi: 10.5271/sjweh.3891.

Schiöler, L. *et al.* (2015) ‘Psychosocial work environment and risk of ischemic stroke and coronary heart disease: A prospective longitudinal study of 75 236 construction workers’, *Scandinavian Journal of Work, Environment and Health*, 41(3), pp. 280–287. doi: 10.5271/sjweh.3491.

Schnall, P. L. *et al.* (1990) ‘The relationship between “job strain”, workplace diastolic blood pressure, and left ventricular mass index: Results of a case-control study’, *Journal of the American Medical Association*. P.L. Schnall, Hypertension Center, Cornell University, Medical College, 525 E 68th St, New York, NY 10021, United States: American Medical Association (515 North State Street, Chicago IL 60654, United States), 263(14), pp. 1929–1935. doi: http://dx.doi.org/10.1001/jama.263.14.1929.

Siegrist, J. *et al.* (1990) ‘Low status control, high effort at work and ischemic heart disease: Prospective evidence from blue-collar men’, *Social Science and Medicine*, 31(10), pp. 1127–1134. doi: 10.1016/0277-9536(90)90234-J.

Slopen, N. *et al.* (2012) ‘Job strain, job insecurity, and incident cardiovascular disease in the women’s health study: Results from a 10-year prospective study’, *PLoS ONE*, 7(7). doi: 10.1371/journal.pone.0040512.

Tenkanen, L. *et al.* (1997) ‘Shift work, occupation and coronary heart disease over 6 years of follow-up in the Helsinki Heart Study’, *Scandinavian Journal of Work, Environment and Health*, 23(4), pp. 257–265. doi: 10.5271/sjweh.218.

Theorell, T. *et al.* (1998) ‘Decision latitude, job strain, and myocardial infarction: a study of working men in Stockholm. The SHEEP Study Group. Stockholm Heart epidemiology Program.’, *American Journal of Public Health*, 88(3), pp. 382–388. doi: 10.2105/ajph.88.3.382.

Tochsen, F. (1993) ‘Working hours and ischaemic heart disease in danish men: A 4-year cohort study of hospitalization’, *International Journal of Epidemiology*, 22(2), pp. 215–221. doi: 10.1093/ije/22.2.215.

Tong, J. *et al.* (2017) ‘Effect of Interaction between Noise and A1166C Site of AT1R Gene Polymorphism on Essential Hypertension in an Iron and Steel Enterprise Workers’, *Journal of Occupational and Environmental Medicine*, 59(4), pp. 412–416. doi: 10.1097/JOM.0000000000000970.

Tsutsumi, A. *et al.* (2009) ‘Prospective study on occupational stress and risk of stroke’, *Archives of Internal Medicine*, 169(1), pp. 56–61. doi: 10.1001/archinternmed.2008.503.

Tsutsumi, A., Kayaba, K. and Ishikawa, S. (2011) ‘Impact of occupational stress on stroke across occupational classes and genders’, *Social Science and Medicine*. Elsevier Ltd, 72(10), pp. 1652–1658. doi: 10.1016/j.socscimed.2011.03.026.

Valery, G. *et al.* (2016) ‘Myocardial infarction and stroke: 16-year risk and stress at work in open population of 25-64-year-old women in Russia/Siberia (who Monica-psychosocial program)’, *Russian Journal of Cardiology*, 132(4), pp. 135–139. doi: 10.15829/1560-4071-2016-4-eng-135-139.

Vetter, C. *et al.* (2016) ‘Association between rotating night shiftwork and risk of coronary heart disease among women’, *JAMA - Journal of the American Medical Association*, 315(16), pp. 1726–1734. doi: 10.1001/jama.2016.4454.

Virkkunen, H. *et al.* (2006) ‘The triad of shift work, occupational noise, and physical workload and risk of coronary heart disease’, *Occupational and Environmental Medicine*, 63(6), pp. 378–386. doi: 10.1136/oem.2005.022558.

Virkkunen, H., Kauppinen, T. and Tenkanen, L. (2005) ‘Long-term effect of occupational noise on the risk of coronary heart disease’, *Scandinavian Journal of Work, Environment and Health*, 31(4), pp. 291–299. doi: 10.5271/sjweh.885.

Virtanen, M. *et al.* (2010) ‘Overtime work and incident coronary heart disease: The Whitehall II prospective cohort study’, *European Heart Journal*, 31(14), pp. 1737–1744. doi: 10.1093/eurheartj/ehq124.

Virtanen, S. V. and Notkol, V. (2002) ‘Socioeconomic inequalities in cardiovascular mortality and the role of work: A register study of Finnish men’, *International Journal of Epidemiology*, 31(3), pp. 614–621. doi: 10.1093/ije/31.3.614.

Wamala, S. P. *et al.* (2000) ‘Job stress and the occupational gradient in coronary heart disease risk in women: The Stockholm Female Coronary Risk Study’, *Social Science and Medicine*, 51(4), pp. 481–489. doi: 10.1016/S0277-9536(00)00006-X.

Wang, A. *et al.* (2016) ‘Shift work and 20-year incidence of acute myocardial infarction: Results from the Kuopio Ischemic Heart Disease Risk Factor Study’, *Occupational and Environmental Medicine*, 73(9), pp. 588–594. doi: 10.1136/oemed-2015-103245.

Yong, M. *et al.* (2014) ‘Shift work and risk of non-cancer mortality in a cohort of German male chemical workers’, *International Archives of Occupational and Environmental Health*, 87(7), pp. 763–773. doi: 10.1007/s00420-013-0922-5.

Zayeri, F., Amini, M. and Hasanzadeh, H. (2018) ‘Assessment of relationship between shift work and hypertension in Mahshahr petrochemical Ataff: A longitudinal study’, *Iranian Journal of Epidemiology*. M. Amini, Department of Biostatistics, Faculty of Medical Sciences, Tarbiat Modares University, Tehran, Iran, Islamic Republic of. E-mail: maedeh.amini@modares.ac.ir: Iranian Epidemiological Association (E-mail: irea.journal@gmail.com), 13(4), pp. 318–327. Available at: http://irje.tums.ac.ir/index.php?&slct_pg_id=10&sid=1&slc_lang=en.
